# Differential expression of the MYC‐Notch axis drives divergent responses to the front‐line therapy in central and peripheral extensive‐stage small‐cell lung cancer

**DOI:** 10.1002/mco2.70112

**Published:** 2025-02-18

**Authors:** Libo Luo, Rui Xia, Shiqi Mao, Qian Liu, He Du, Tao Jiang, Shuo Yang, Yan Wang, Wei Li, Fei Zhou, Jia Yu, Guanghui Gao, Xuefei Li, Chao Zhao, Lei Cheng, Jingyun Shi, Xiaoxia Chen, Caicun Zhou, Luonan Chen, Shengxiang Ren, Fengying Wu

**Affiliations:** ^1^ Department of Medical Oncology Shanghai Pulmonary Hospital Tongji University School of Medicine Shanghai China; ^2^ Key Laboratory of Systems Biology Center for Excellence in Molecular Cell Science Shanghai Institute of Biochemistry and Cell Biology Chinese Academy of Sciences Shanghai China; ^3^ University of Chinese Academy of Sciences Beijing China; ^4^ Department of Oncology Xiangyang No. 1 People's Hospital Hubei University of Medicine Xiangyang China; ^5^ Department of Radiology Shanghai Pulmonary Hospital Tongji University School of Medicine Shanghai China; ^6^ Department of Lung Cancer and Immunology Shanghai Pulmonary Hospital Tongji University School of Medicine Shanghai China; ^7^ Key Laboratory of Systems Health Science of Zhejiang Province School of Life Science Hangzhou Institute for Advanced Study University of Chinese Academy of Sciences Chinese Academy of Sciences Hangzhou China

**Keywords:** chemo‐immunotherapy, extensive‐stage small‐cell lung cancer, primary tumor location, single‐cell RNA sequencing

## Abstract

Central and peripheral extensive‐stage small‐cell lung cancer (ES‐SCLC) are reported to be two distinct tumor entities, but their responses to the front‐line therapies and underlying biological mechanisms remain elusive. In this study, we first compared the outcomes of central and peripheral ES‐SCLC receiving front‐line chemotherapy or chemo‐immunotherapy with a cohort of 265 patients. Then we performed single‐cell RNA sequencing (scRNA‐seq) on nine treatment‐naïve ES‐SCLC samples to investigate potential mechanisms underlying the response differences. Under chemotherapy, the peripheral type had a lower objective response rate (44.8% vs. 71.2%, *p *= 0.008) and shorter progression‐free survival (median 3.4 vs. 5.1 months, *p *= 0.001) than the central type. When comparing chemo‐immunotherapy with chemotherapy, the peripheral type showed a greater potential to reduce progression (HR, 0.18 and 0.52, respectively) and death (HR, 0.44 and 0.91 respectively) risks than the central type. Concerning the scRNA‐seq data, the peripheral type was associated with chemo‐resistant and immune‐responsive tumoral and microenvironmental features, including a higher expression level of MYC‐Notch‐non‐neuroendocrine (MYC‐Notch‐non‐NE) axis and a more potent antigen presentation and immune activation status. Our results revealed that central and peripheral ES‐SCLC had distinct responses to front‐line treatments, potentially due to differential activation statuses of the MYC‐Notch‐non‐NE axis.

## Graphical abstract

This study found that peripheral ES‐SCLC was more chemo‐resistant and ICI‐responsive in the front‐line setting compared with the central type. The activation of the MYC‐Notch axis and downregulation of the inhibitory Notch signaling ligand DLL3 in the peripheral type may explain the response differences.

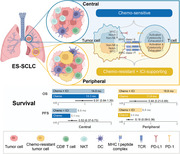

## 1 INTRODUCTION

Small‐cell lung cancer (SCLC) accounts for 10%–15% of all lung cancer cases and is characterized by early metastatic dissemination, rapid tumor growth, and dismal prognosis.^1^, ^2^, ^3^ Currently, accumulating evidence from IMpower133,^4^ CASPIAN,^5^ ASTRUM‐005,^6^ and CAPSTONE‐1^7^ trials consistently suggested that the front‐line standard of care has switched from platinum‐based chemotherapy to the addition of immune checkpoint inhibitor (ICI) in patients with extensive‐stage SCLC (ES‐SCLC).^8^, ^9^ However, the overall survival benefit from chemo‐immunotherapy is moderate and its efficacy biomarkers remain largely unknown even though tremendous efforts have been invested in this field.^10^, ^11^, ^12^, ^13^, ^14^, ^15^ Hence, it is imperative to identify the potential population benefit from these regimens.

Based on the primary location, SCLC is categorized into central and peripheral types, with the former involving segmental or more proximal bronchi and the latter affecting sub‐segmental or more distal bronchi.^16^, ^17^ Previous reports indicated that central and peripheral SCLC exhibited differences in clinicopathological features, genomic characteristics, and prognosis.^16^, ^18^, ^19^, ^20^, ^21^, ^22^ Peripheral type was associated with a higher prevalence of interstitial lung disease,^18^ lower PD‐L1 expression,^20^ higher tumor mutation burden (TMB),^21^ and increased thyroid transcription factor‐1 (TTF1) expression.^22^ Central and peripheral SCLC also differ in prognosis, with reports showing inconsistent results about which type has better survival.^16^, ^18^, ^19^, ^21^, ^22^ However, all these studies included a substantial proportion of limited‐stage SCLC (LS‐SCLC), and the heterogeneity between central and peripheral ES‐SCLC is still not fully elucidated. Despite this, these pieces of evidence support the notion that the two types of ES‐SCLC may hold the promise to predict responses to front‐line therapy.

Recent advances in transcriptomic testing have revolutionized the classification of SCLC and shed light on its precision medicine.^23^, ^24^, ^25^, ^26^ Rudin et al.^23^ first synthesized previous lines of evidence and proposed a nomenclature of SCLC subtypes defined by the expression of four transcription factors (TFs), dubbed ASCL1, NEUROD1, POU2F3, and YAP1. Thereafter, Gay et al.^26^ also identified four SCLC subtypes via unbiased clustering of RNA‐seq data from SCLC resections, including three defined by Rudin et al.^23^ (SCLC‐A, SCLC‐N, and SCLC‐P) and a fourth one characterized by an inflamed gene signature (SCLC‐I). Of note, SCLC‐I was found to be associated with superior outcomes to chemo‐immunotherapy.^26^ Meanwhile, scRNA‐seq technologies enable detailed dissection of tumor ecosystems in single‐cell resolution and have been amply leveraged in patients with lung cancer.^27^, ^28^ Previous reports, including ours, have mapped the transcriptomic landscapes of tumor cells and their microenvironment of advanced non‐small cell lung cancer by scRNA‐seq.^29^, ^30^, ^31^, ^32^ scRNA‐seq has also been successfully applied in exploring response to immunotherapy.^27^, ^33^ With regards to SCLC, which has been perceived as a representative cancer with high lineage plasticity,^34^ scRNA‐seq analyses are scant and limited to early‐staged or posttreatment cases.^35^, ^36^, ^37^, ^38^ The transcriptomic landscapes of central and peripheral ES‐SCLC at baseline and the association with responses to front‐line therapy are not yet well‐defined.

In this study, we first collected a clinical cohort of 265 patients to compare the responses of central and peripheral ES‐SCLC to front‐line treatments, including chemotherapy and chemo‐immunotherapy. Then we profiled and compared the baseline scRNA‐seq characteristics of central and peripheral ES‐SCLC to identify potential mechanisms underlying the response discrepancy and validate the findings with clinical and transcriptomic data from the IMpower133 trial. We found that the peripheral type was associated with an inferior response to front‐line chemotherapy while experiencing more survival benefit from the addition of PD‐1/PD‐L1 blockade to chemotherapy compared with the central type. Mechanistically, the activation of the MYC‐Notch‐non‐NE axis in the peripheral type might account for the response differences.

## 2 RESULTS

### 2.1 Patients’ characteristics

**FIGURE 1:**
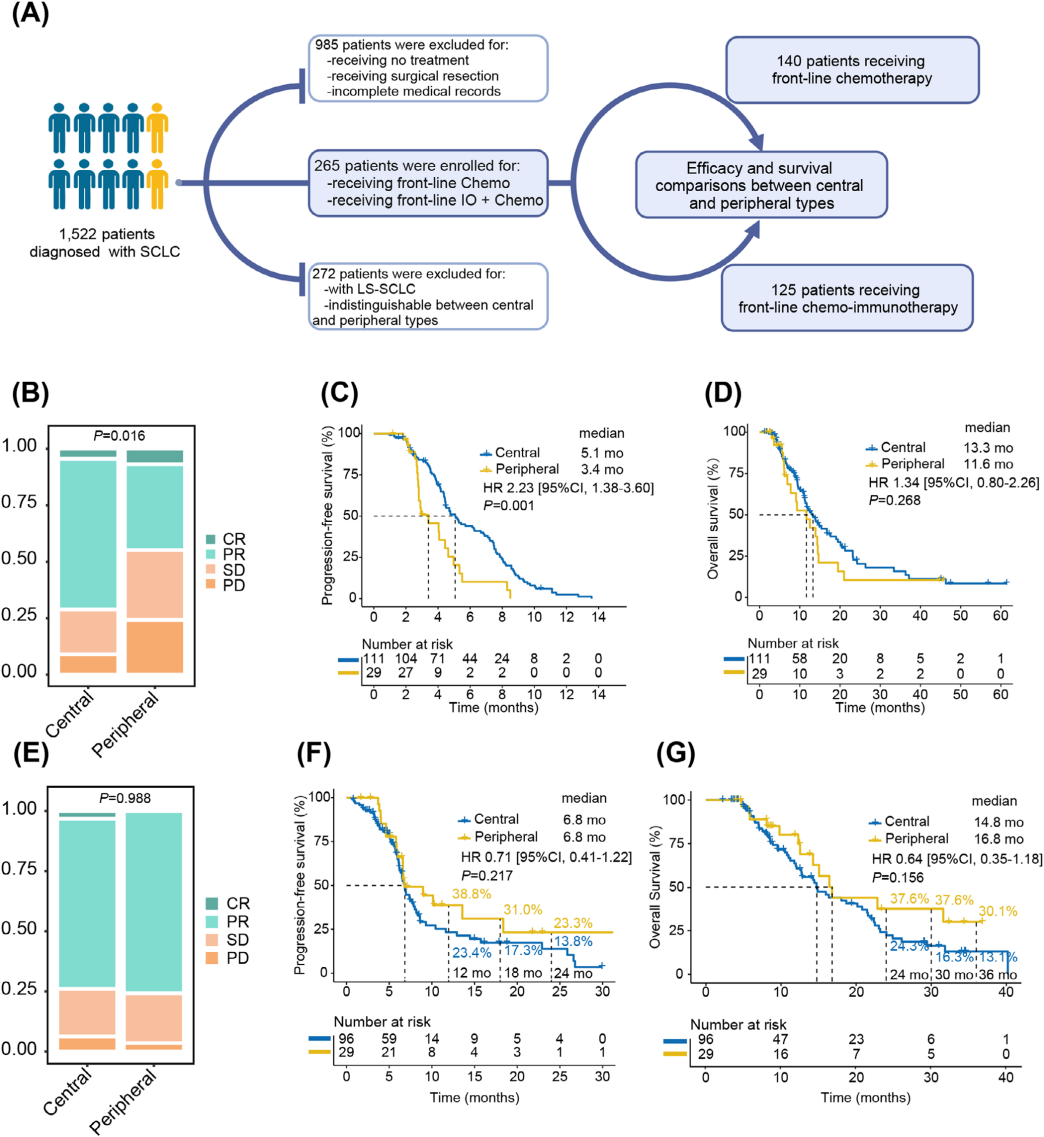
Central and peripheral ES‐SCLC responded differently to the front‐line treatments. (A) Flow chart depicting the process of patient screening. (B) Stacked bar chart for best overall response and survival curves for (C) progression‐free survival or (D) overall survival in patients treated with chemotherapy (*n* = 111 and 29 patients for central and peripheral types, respectively). (E) Stacked bar chart for best overall response and survival curves for (F) progression‐free survival or (G) overall survival in patients treated with chemo‐immunotherapy (*n* = 96 and 29 patients for central and peripheral types, respectively). CR, complete response; HR, hazard ratio; LS‐SCLC, limited‐stage SCLC; PD, progressive disease; PR, partial response; SCLC, small‐cell lung cancer; SD, stable disease.

A total of 1522 patients histologically diagnosed as SCLC at Shanghai Pulmonary Hospital were identified. By carefully reviewing clinicopathologic data, 985 patients receiving no treatment or surgical resection or with incomplete medical records and 272 patients with LS‐SCLC or uncertain primary tumor location were excluded. Finally, 265 patients with ES‐SCLC who received either front‐line chemotherapy or chemo‐immunotherapy were enrolled (Figure 1A). Among them, 207 (78.1%) and 58 (21.9%) were central and peripheral types; 140 and 125 received chemotherapy and chemo‐immunotherapy, respectively. The chemotherapy group comprised 111 (79.3%) central type and 29 (20.7%) peripheral type patients, and the chemo‐immunotherapy group was made up of 96 (76.8%) and 29 (23.2%) central and peripheral patients, correspondingly. The baseline characteristics and detailed treatment information were presented in Tables S1 and S2, respectively.

### 2.2 Efficacy of the chemotherapy group

The objective response rate (ORR) was 65.7% and the disease control rate (DCR) was 87.8% in the chemotherapy group. The peripheral type exhibited a significantly inferior ORR (44.8% vs. 71.2%, *p *= 0.008) and DCR (75.9% vs. 91.0%, *p *= 0.026) than the central type (Table S3). The nonparametric rank‐sum test also confirmed the inferior response of the peripheral type (*p *= 0.016; Figure 1B). Detailed analyses of the response data revealed that the peripheral type had a significantly lower PR rate (37.9% vs. 66.7%, *p *= 0.005) and a marginally higher PD rate (24.2% vs. 9.0%, *p *= 0.057) compared with the central type (Table S3).

The overall median progression‐free survival (PFS) and overall survival (OS) were 4.6 and 12.9 months, respectively. The peripheral type was associated with a significantly shorter PFS (median 3.4 vs. 5.1 months, *p *= 0.001) and a numerically shorter OS (median 11.6 vs. 13.3 months, *p *= 0.268; Figures 1C,D). Furthermore, the peripheral type was associated with inferior PFS in most subgroups (Figure S1).

Univariate analyses indicated that liver (HR: 1.79, 95% CI: 1.10–2.92, *p *= 0.019) and bone (HR: 1.66, 95% CI: 1.13–2.42, *p *= 0.009) metastases, as well as peripheral type (HR: 2.23, 95% CI: 1.38–3.60, *p *= 0.001), were significant risk factors for progression (Table S4). This significance persisted in the multivariate model for liver (HR: 1.91, 95% CI: 1.14–3.20, *p *= 0.013) and bone (HR: 1.48, 95% CI: 1.00–2.18, *p *< 0.050) metastases, along with peripheral type (HR: 2.41, 95% CI: 1.46–3.99, *p *< 0.001). For OS, bone metastasis (HR: 1.49, 95% CI: 0.96–2.32, *p *= 0.073) was identified as a marginally significant risk factor in the univariate model.

### 2.3 Efficacy of the chemo‐immunotherapy group

In the chemo‐immunotherapy group, the ORR and DCR for all patients were 74.4% and 94.4%, respectively. The two types of ES‐SCLC demonstrated comparable ORR (75.9% vs. 74.0%, *p *= 0.837) and DCR (96.6% vs. 93.8%, *p *= 0.909; Table S3). The nonparametric rank‐sum test also showed no statistical difference in best overall response (Figure 1E).

The median PFS and OS were 6.8 and 15.1 months, respectively. Peripheral and central types had similar median PFS (6.8 vs. 6.8 months, *p *= 0.217) and median OS (16.8 vs. 14.8 months, *p *= 0.156; Figures 1F,G). Nevertheless, the peripheral type was consistently associated with numerically higher 1‐ (38.8% vs. 23.4%, *p *= 0.664), 1.5‐ (31.0% vs. 17.3%, *p *= 0.763), and 2‐year (23.3% vs. 13.8%, *p *= 0.871) PFS rates, together with 2‐ (37.6% vs. 24.3%, *p *= 0.731), 2.5‐ (37.6% vs. 16.3%, *p *= 0.627), and 3‐year OS rates (30.1% vs. 13.1%, *p *= 0.752; Figures 1F,G). The results of subgroup analyses and Cox regression analyses are given in Figure S2 and Table S4.

### 2.4 Outcome comparison of chemo‐immunotherapy with chemotherapy in patients with central or peripheral ES‐SCLC

**FIGURE 2:**
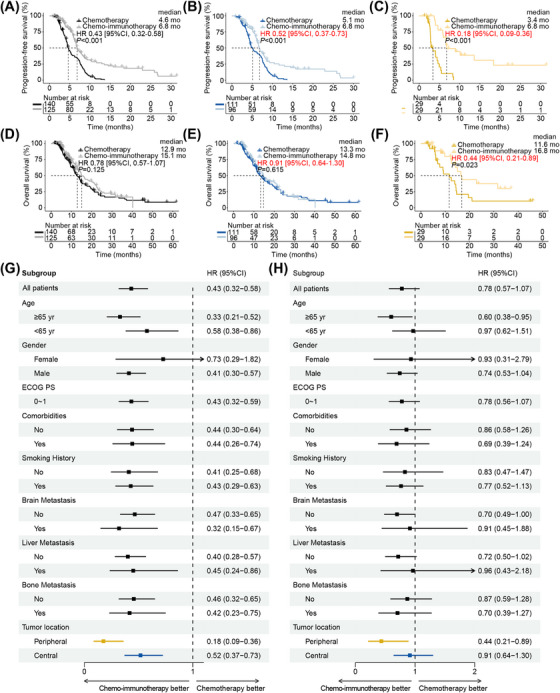
Peripheral ES‐SCLC benefited more from the addition of PD‐1/PD‐L1 blockade to chemotherapy. Survival curves for progression‐free survival in (A) total patients, (B) central type patients, or (C) peripheral type patients. Survival curves for overall survival in (D) total patients, (E) central type patients, or (F) peripheral type patients; Forest plots of subgroup analysis for (G) progression‐free survival or (H) overall survival in total patients. HR, hazard ratio; ECOG PS, Eastern Corporation Oncology Group performance status.

As chemo‐immunotherapy has become the standard front‐line treatment for ES‐SCLC, we further compared its performance with that of chemotherapy. The baseline characteristics were generally balanced (Table S1). The total patients (6.8 vs. 4.6 months, *p *< 0.001), central patients (6.8 vs. 5.1 months, *p *< 0.001), and peripheral patients (6.8 vs. 3.4 months, *p *< 0.001) all demonstrated significantly longer PFS when receiving chemo‐immunotherapy (Figures 2A–C). Notably, the peripheral type was associated with a greater decrease in progression risk than the central type (HR [95% CI], 0.18 [0.09, 0.36] vs. 0.52 [0.37, 0.73]) (Figures 2B,C,G). Meanwhile, the peripheral type (16.8 vs. 11.6 months, *p *= 0.023) demonstrated significant overall survival improvement when receiving chemo‐immunotherapy, while the central type (14.8 vs. 13.3 months, *p *= 0.615) only showed numerical prolongation (Figures 2D–F). Likewise, peripheral type was also correlated with a more pronounced reduction of mortality risk than central type (HR [95% CI], 0.44 [0.21, 0.89] vs. 0.91 [0.64, 1.30]; Figures 2E,F,H).

### 2.5 Overview of the scRNA‐seq data in central and peripheral ES‐SCLC

**FIGURE 3:**
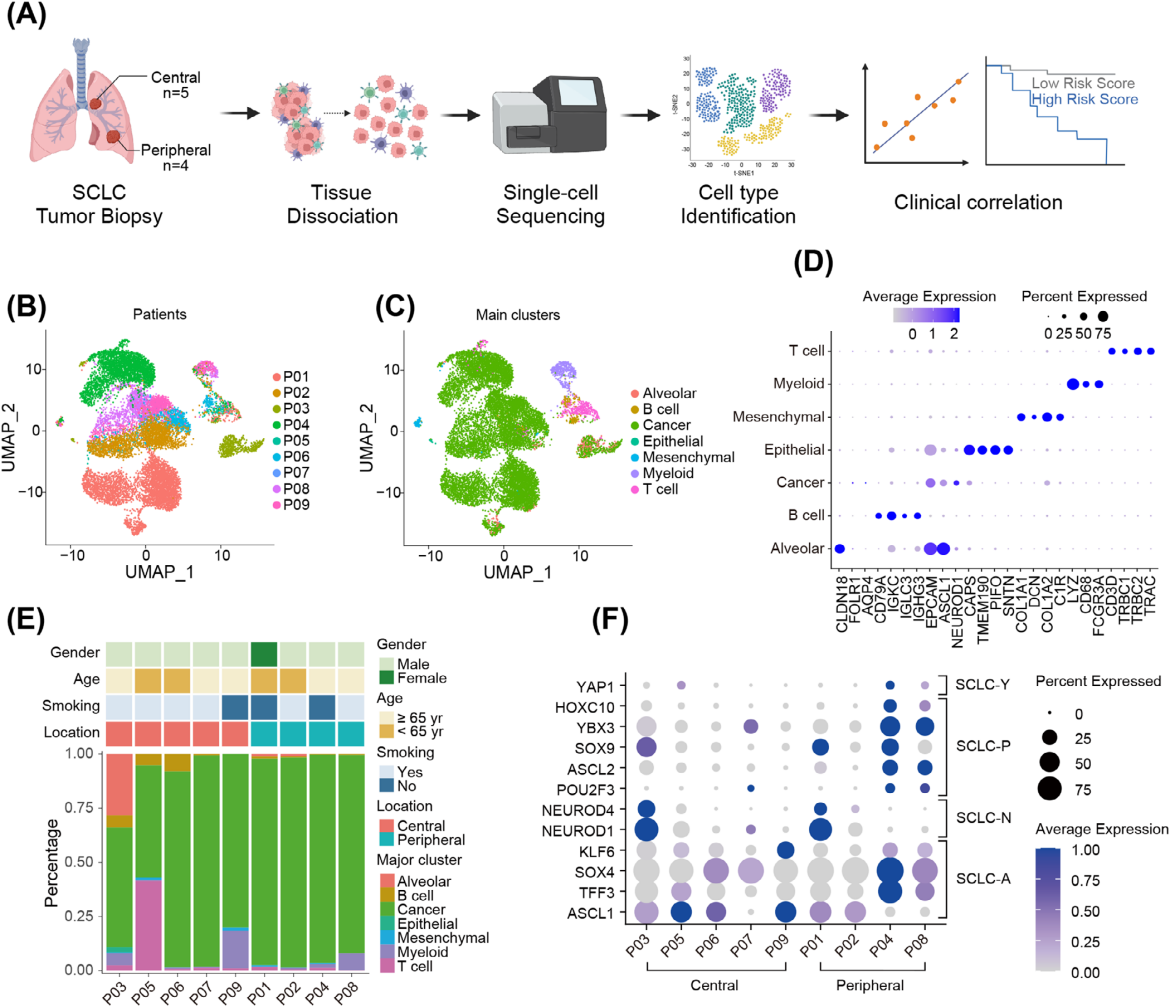
Overview of the scRNA‐seq data. (A) Experimental design of scRNA‐seq; UMAP plots of 12,897 cells from nine patients, colored by (B) patients or (C) seven major clusters. (D) Expression of marker genes for major clusters in (C). (E) Cell type composition and clinical information of each patient. (F) Expression of classical subtype‐specific transcription factors of each patient. SCLC, small‐cell lung cancer; ES‐SCLC, extensive‐stage SCLC; UMAP, uniform manifold approximation and projection. SCLC‐Y, SCLC‐P, SCLC‐N, and SCLC‐A represent subtypes of small‐cell lung cancer that highly express the transcription factors YAP1, POU2F3, NEUROD1, and ASCL1, respectively.

To uncover the mechanisms underlying the distinct responses to front‐line therapy of central and peripheral ES‐SCLC, scRNA‐seq was conducted with biopsy samples from nine treatment‐naïve patients. Among them, four patients were of peripheral type and five of central type (Figure 3A). Detailed clinicopathologic information of the nine patients has been summarized in Table S5. A total of 18,434 cells were identified and 12,897 cells were reserved for downstream analyses after quality control and cell filtering (Figure 3B). These cells were clustered and annotated into seven coarse cell types, dubbed cancer cells, T cells, myeloid cells, B cells, mesenchymal cells, alveolar cells, and normal epithelial cells (Figure 3C). Specifically expressed marker genes of these clusters were displayed in Figure 3D. Cancer cells dominated cell composition in every sample examined and the proportions of the cell types varied considerably among samples, indicating intrinsic interindividual heterogeneity (Figure 3E). Notably, we found that the cell type composition of central and peripheral ES‐SCLC was disparate (Figure 3E). To be specific, the proportions of tumor cells in the peripheral type were higher and relatively uniform among patients, whereas in the central type, they were lower and varied among patients. In addition, when profiling the expression of TFs^23,^ ^37^ defining four SCLC subtypes, we observed evident co‐expression of these TFs within a single sample and obvious discrepancy between central and peripheral types (Figure 3F). We found that central and peripheral types expressed similar levels of SCLC‐A and SCLC‐N TFs, while peripheral type also showed high expression of SCLC‐P and SCLC‐Y TFs (Figure 3F). These evidence suggested that central and peripheral ES‐SCLC had distinct tumoral and microenvironmental properties.

### 2.6 Peripheral ES‐SCLC was associated with the dysfunction of chemo‐resistance‐related genes and pathways

As we observed that peripheral ES‐SCLC showed an inferior response to chemotherapy (Figures 1B–D; Figure S1; Table S3), we aimed to investigate whether there were differences in the activation of chemo‐resistance‐related genes and pathways between central and peripheral types.

**FIGURE 4:**
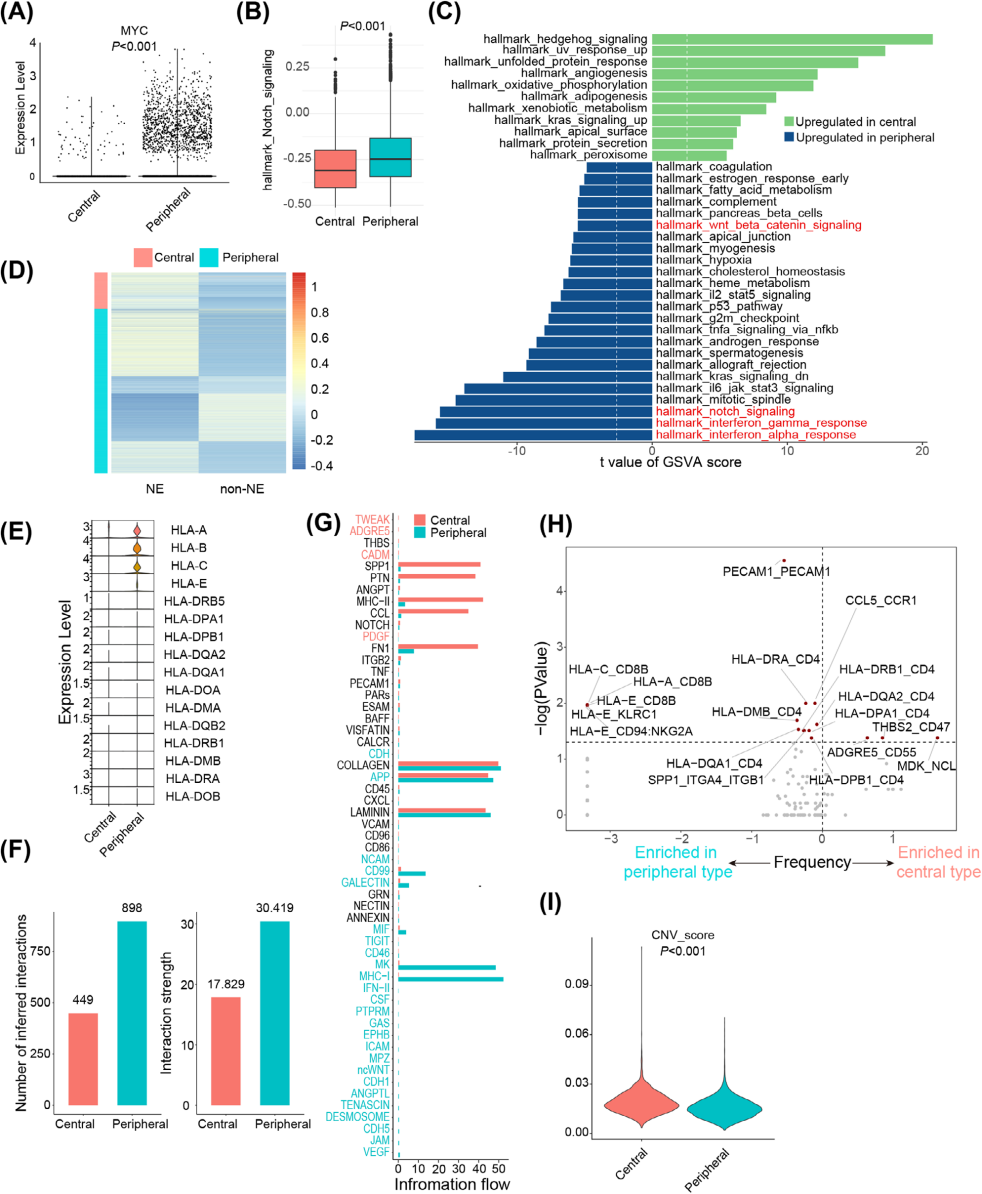
Distinct tumoral characteristics of central and peripheral ES‐SCLC. (A) MYC expression levels in each cancer cell. (B) Cell activity scores of Notch signaling for each cancer cell. (C) Differences in Hallmark pathway activities scored per cell by GSVA between central and peripheral cancer cells, and the vertical white dotted lines denote critical T values with adjusted *p*‐values of 0.05. (D) Cell activity scores of NE and non‐NE signatures for each cancer cell. (E) Expression levels of HLA genes. (F) Number of inferred interactions (left) and interaction strength (right). (G) Inferred interaction pathways for central and peripheral ES‐SCLC. (H) Differential ligand/receptor pairs (central type versus peripheral type). (I) CNA scores of central and peripheral tumor cells. For (A–C) and (I), *n* = 2124 and 9594 cancer cells for central and peripheral types, respectively. CNA, copy number alteration; ES‐SCLC, extensive‐stage SCLC; GSVA, gene set variation analysis; NE, neuroendocrine; non‐NE, non‐neuroendocrine.

In general, 11,718 cancer cells were investigated, among which 2124 and 9594 were from central and peripheral ES‐SCLC, correspondingly (Figures S3A,B). We first reviewed the differentially expressed genes (DEGs) list between central and peripheral ES‐SCLC concentrating on previously reported chemo‐resistance‐related genes.^39^ We found that the peripheral type was associated with a higher expression level of MYC and a lower expression level of SLFN11 (Figure 4A; Figures S3C–E). MYC overexpression and low SLFN11 expression are previously reported to be associated with SCLC chemo‐resistance.^39^, ^40^, ^41^, ^42^ Notably, MYC has been found to dedifferentiate neuroendocrine (NE) SCLC into non‐neuroendocrine (non‐NE) subtypes through the activation of Notch signaling.^40^ And both Notch signaling activation and non‐NE differentiation were reported to mediate SCLC chemo‐resistance.^39^, ^43^

We then compared the expression levels of Notch signaling and the non‐NE differentiation statutes of central and peripheral ES‐SCLC. Using gene set variation analysis (GSVA) on all genes expressed in the included tumor cells, we found that tumor cells from the peripheral type exhibited upregulated Notch signaling, along with activation of Wnt/β‐catenin signaling, interferon α response, and interferon γ response compared with the central type (Figures 4B,C; Figure S3F). Further, we investigated the expression of NE and non‐NE gene signatures^44^ of tumor cells and found that the central type was mainly associated with NE differentiation, while a substantial proportion of peripheral tumor cells showed non‐NE differentiation (Figure 4D).

In summary, these findings suggest that tumor cells from peripheral ES‐SCLC had a higher expression level of the MYC‐Notch‐non‐NE axis, which might contribute to its chemo‐resistant properties to a significant extent.

### 2.7 Peripheral ES‐SCLC was linked to an immune‐responsive phenotype

In the clinical cohort, we found that peripheral ES‐SCLC responded poorly to front‐line chemotherapy while driving more benefit from chemo‐immunotherapy than central type (Figures 1B–G, 2; Figures S1, S2; Table S3). Besides, the peripheral type showed upregulated interferon α/γ responses (Figure 4C). We considered that the two types may also differ in the ability to activate anti‐tumor immunity. Then, we further compared their microenvironment phenotypes.

We first conducted gene ontology (GO) enrichment analysis of differentially expressed genes of cancer cells and found significant enrichment of GO term “presentation of endogenous peptide antigen via MHC class I” (Figure S3G), indicating discrepancy in antigen presentation and immune activation. We next compared the expression levels of HLA genes in the two types and noticed that the expression of MHC class I genes (HLA‐A, HLA‐B, and HLA‐C) was significantly upregulated in the peripheral type (Figure 4E). Further, we performed intercellular interaction analysis and found that the peripheral type was associated with a larger number of interactions and a higher level of interaction strength (Figure 4F). Intercellular interactions in the peripheral type were mainly through pathways like MHC‐I and MK and via pathways like SPP1, PTN, MHC‐II, and CCL in the central type (Figure 4G). Differential ligand/receptor pairs between central and peripheral types were presented in Figure 4H, which also suggested that tumor cells of peripheral ES‐SCLC might frequently interact with other cells through HLA molecules. Besides, peripheral‐type tumor cells demonstrated significantly lower copy number alteration (CNA) scores than central type (Figure 4I; Figure S3H). CNA was reported to be a negative efficacy biomarker for PD1/PD‐L1 blockade.^45^ Overall, the peripheral type showed more potent ability in antigen presentation and immune activation.

**FIGURE 5:**
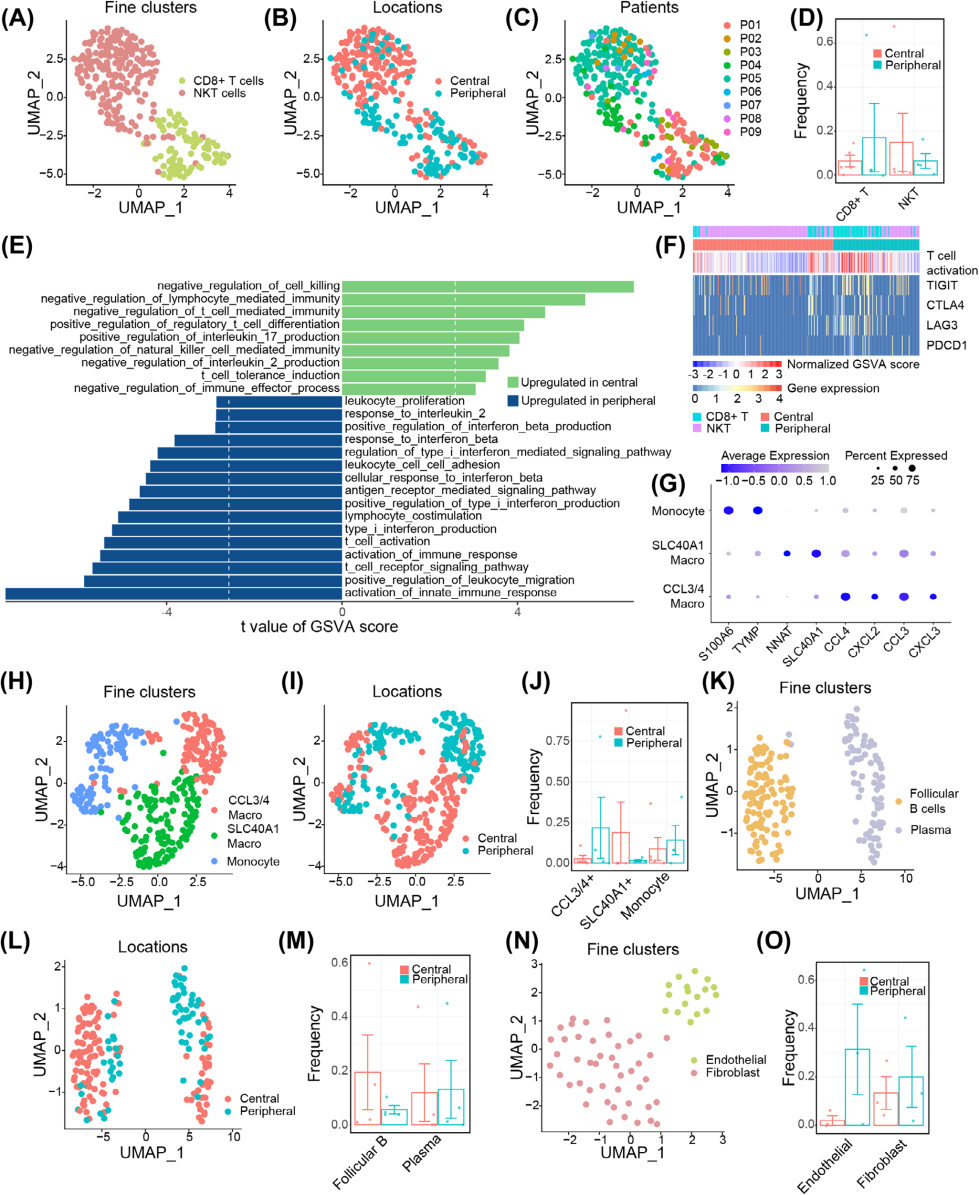
Divergent microenvironmental landscapes of central and peripheral ES‐SCLC. UMAP plots of T cells, colored by (A) subclusters, (B) primary tumor locations, or (C) patients. (D) Percentages of each T‐cell subcluster in total T cells of central and peripheral type ES‐SCLC (*n* = 5 and 4 for central and peripheral types, respectively). (E) Differences in Hallmark pathway activities scored per cell by GSVA between central and peripheral T cells, the vertical white dotted lines denote critical *T* values with adjusted *p*‐values of 0.05 (*n* = 185 and 114 T cells for central and peripheral types, respectively). (F) Heatmap depicting cell activity on T cell activation and expression of immune checkpoint genes. (G) Expression levels of marker genes of three Myeloid subclusters; UMAP plots of Myeloid cells, colored by (H) subclusters or (I) primary tumor locations. (J) Percentages of each Myeloid subcluster in total Myeloid cells of central and peripheral ES‐SCLC (*n* = 5 and 4 for central and peripheral types, respectively); UMAP plots of B cells, colored by (K) subclusters or (L) primary tumor locations. (M) Percentages of each B cell subcluster in total B cells of central and peripheral ES‐SCLC (*n* = 5 and 4 for central and peripheral types, respectively). (N) UMAP plot of mesenchymal cells, colored by subclusters. (O) Percentages of each mesenchymal cell subcluster in total mesenchymal cells of central and peripheral ES‐SCLC (*n* = 5 and 4 for central and peripheral types, respectively). ES‐SCLC, extensive‐stage SCLC; GSVA, gene set variation analysis; UMAP, uniform manifold approximation and projection.

To further delineate the nonmalignant cell landscapes of the two ES‐SCLC types, we compared the cell types individually. 299 T cells were identified and clustered into two subclusters, CD8^+^ T cells and NKT cells (Figures 5A–C; Figure S4A). The peripheral type was associated with a numerically higher percentage of infiltrating CD8^+^ T cells, whereas the central type was prone to be infiltrated with NKT cells (Figure 5D). GO enrichment analysis of DEGs showed that T cells from the two types of ES‐SCLC exhibited differences in pathways related to immune response and immune cell differentiation (Figure S4B). Additional GSVA analyses showed that T cells from peripheral ES‐SCLC demonstrated upregulated pathways concerning cytokine production, immune cell activation, immune response, TCR signaling, and cytotoxicity; while T cells from the central type mainly enriched in pathways that negatively regulate immunity or positively contribute to immune tolerance (Figures 5E,F). T cells of the peripheral type also showed high expression of immune checkpoint molecules (Figure 5F). A total of 357 myeloid cells were recorded and further clustered into three subtypes, including monocytes, CCL3/4^+^ macrophages, and SLC40A1^+^ macrophages (Figures 5G–I). SLC40A1^+^ and CCL3/4^+^ macrophages accounted for the most myeloid cells in central and peripheral types, respectively (Figure 5J). CCL3/4 were reported to facilitate the infiltrating of inhibitive CCR5^+^ MDSCs, and concurrent blocking CCR5/CCR5‐ligand and PD1/PD‐L1 axes could enhance efficacy and prolong survival in preclinical tumor models.^46^ Clustering of 187 B cells identified follicular B‐ and plasma cells, and the former was more prevalent in the central type (Figures 5K–M; Figures S5A,B). Likewise, 62 mesenchymal cells were analyzed, and fibroblasts and endothelial cells were recognized (Figures 5N,O; Figure S6).

In brief, compared with the central type, peripheral ES‐SCLC exhibited a more immune‐responsive phenotype, observed in both tumor cells and tumor‐infiltrating lymphocytes.

### 2.8 MYC‐Notch‐non‐NE axis activation status effectively predicted responses to front‐line therapies of ES‐SCLC

Based on the findings from the clinical cohort and scRNA‐seq data, we concluded that peripheral ES‐SCLC was more chemo‐resistant compared with the central type and inferred that the activation status of the chemo‐resistance‐related MYC‐Notch‐non‐NE axis might provide the explanation. We then utilized the clinical and transcriptomic data from IMpower133 trial^4^ to validate our assumption.

**FIGURE 6:**
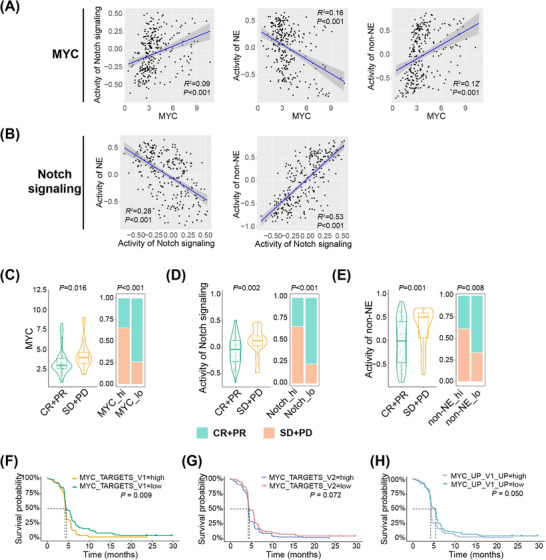
Activation of the MYC‐Notch‐non‐NE axis was associated with chemo‐resistance in the IMpower133 trial. (A) Correlation between MYC expression level and Notch signaling activity (left), NE GSVA score (middle), or non‐NE GSVA score (right). (B) Correlation between Notch signaling activity and NE GSVA score (left) or non‐NE GSVA score (right). Correlation between (C) MYC expression level, (D) Notch signaling activity, or (E) non‐NE GSVA score and response to chemotherapy. Survival curves for progression‐free survival in patients receiving chemotherapy, grouped by the expression level of gene set (F) MYC_TARGETS_V1, (G) MYC_TARGETS_V2, or (H) MYC_UP_V1_UP. In (A, B), *n* = 271; in (C–E), *n* = 134; in (F–H), *n* = 139. CR, complete response; GSVA, gene set variation analysis; NE, neuroendocrine; PD, progressive disease; PR, partial response; SD, stable disease.

We first examined the correlations of expression levels between the components of this axis. We exploited GSVA to evaluate the pathway activity of Notch signaling, NE, and non‐NE differentiation. As expected, the results indicated that MYC expression was significantly positively associated with the activity of Notch signaling (Figure 6A). Meanwhile, both MYC expression and Notch signaling activity exhibited a positive correlation with non‐NE differentiation and a negative correlation with NE differentiation (Figures 6A,B). Furthermore, we found that patients who achieved CR or PR while receiving chemotherapy had lower MYC expression levels, Notch signaling activity, and non‐NE GSVA scores compared with those who achieved SD or PD (Figures 6C–E). Using the median values as cut‐off points, we found that patients with low MYC expression, Notch signaling activity, or non‐NE GSVA scores had higher ORRs (Figures 6C–E). In addition, the expression level of MYC downstream target genes was a negative predictor for PFS (Figures 6F–H). These results together with our findings from central and peripheral ES‐SCLC suggest the chemo‐resistant role of MYC‐Notch‐non‐NE axis in ES‐SCLC.

**FIGURE 7:**
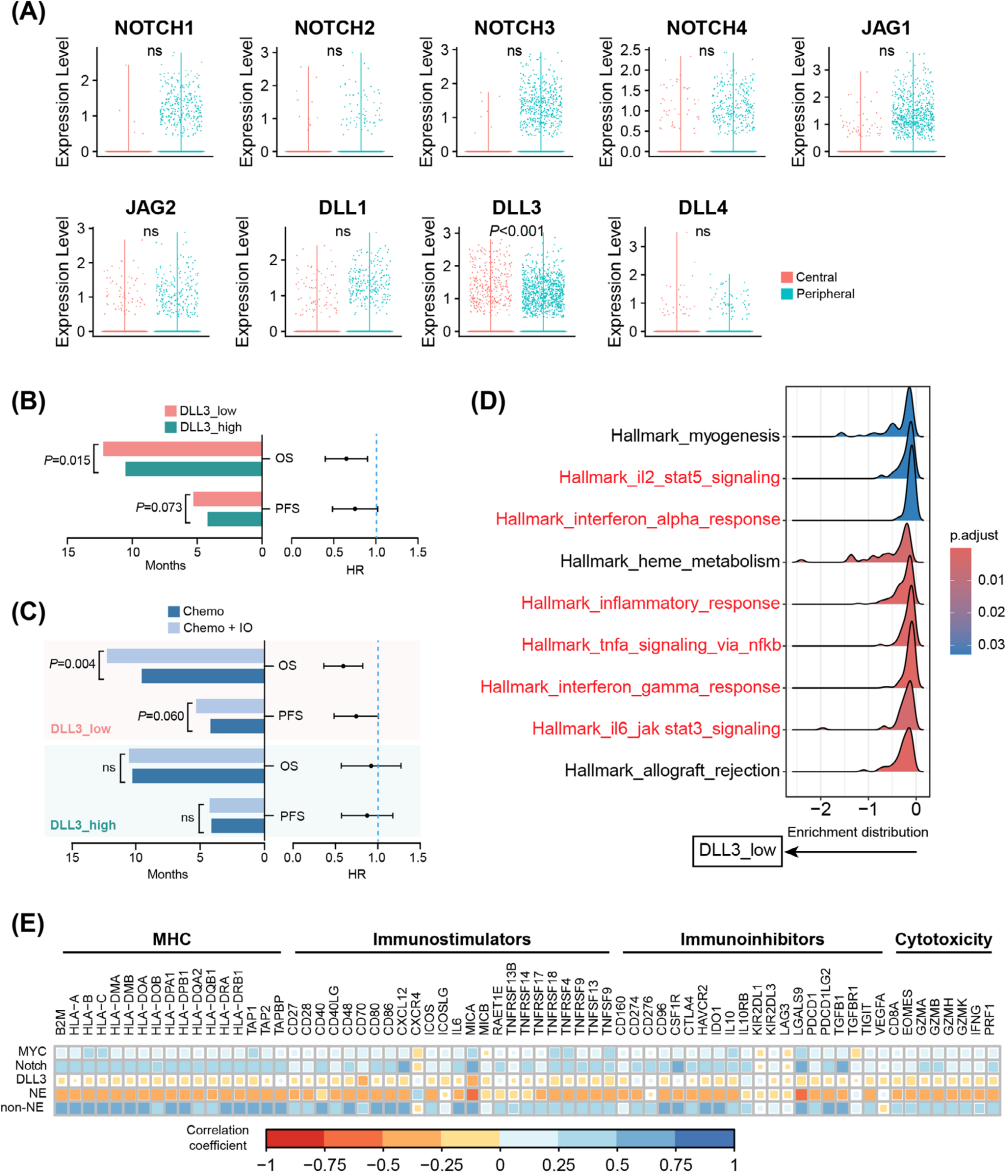
DLL3 expression level effectively predicted the response to chemo‐immunotherapy in the IMpower133 trial. (A) Expression of 4 Notch receptors and 5 ligands in patients with central or peripheral ES‐SCLC. (B) PFS and OS in patients with low or high DLL3 expression levels in the chemo‐immunotherapy group. (C) Comparisons of PFS and OS between patients treated with chemotherapy and those treated with chemo‐immunotherapy, stratified by DLL3 expression level. (D) Ridge plot showing the normalized enrichment score based on GSEA. (E) Heatmap depicting the Pearson correlation of expression levels. In (A), *n* = 2124 and 9594 cancer cells for central and peripheral types, respectively.; in (B), *n* = 132; in (C), *n* = 139 and 132 for the chemotherapy and chemo‐immunotherapy groups, respectively; in (D), *n* = 271. GSEA, gene set enrichment analysis; HR, hazard ratio; NE, neuroendocrine; OS, overall survival; PFS, progression‐free survival.

Recently, NOTCH1 expression was reported to be associated with OS in ES‐SCLC patients treated with atezolizumab.^47^ We hence investigated whether Notch signaling activated in peripheral ES‐SCLC also contributed to its immune‐responsive properties. When profiling the expression levels of the genes encoding the four Notch receptors (NOTCH1‐4) and five ligands (JAG1/2 and DLL1/3/4), we observed no differences in the expression of any genes except for DLL3 between central and peripheral ES‐SCLC (Figure 7A). Peripheral type had a significantly lower DLL3 expression level (Figure 7A). DLL3 is an inhibitory Notch ligand that negatively regulates the signaling pathway and has emerged as a promising target for SCLC treatment recently.^48^ High DLL3 expression was reported to compromise the anti‐tumor immunity and associated with a shorter PFS in a clinical cohort with 30 SCLC patients who received chemo‐immunotherapy.^49^ However, whether DLL3 expression could effectively predict OS remains elusive and the findings by Owen et al.^49^ need to be verified with a bigger sample size. We subsequently evaluated the prediction value of DLL3 with data from the IMpower133 trial. In patients receiving atezolizumab and chemotherapy, low DLL3 expression correlated positively with longer PFS (*p *= 0.073) and OS (*p *= 0.015; Figure 7B). Additionally, patients with low DLL3 expression experienced significant survival benefits in terms of PFS (*p *= 0.060) and OS (*p *= 0.004) when treated with chemo‐immunotherapy compared with chemotherapy alone (Figure 7C). In contrast, patients with high DLL3 expression showed no survival difference between the two treatment approaches (Figure 7C). Gene set enrichment analysis (GSEA) also showed that low DLL3 expression was linked to the activation of immune and inflammatory pathways, such as the interferon α/γ responses (Figure 7D), consistent with the characteristics of peripheral ES‐SCLC (Figure 4C). Besides, we observed that MYC expression, Notch signaling activity, and non‐NE activity each showed broad positive correlations with the expression of MHC, immunostimulatory, immunoinhibitory, and cytotoxic molecules. Conversely, both DLL3 expression and NE activity exhibited clear negative correlations (Figure 7E).

All in all, we found that the activation of the MYC‐Notch‐non‐NE axis was connected to chemo‐resistance in ES‐SCLC, and DLL3, as a key inhibitory component of Notch signaling, served as a potent predictor for identifying patients who would benefit from chemo‐immunotherapy. These findings may explain the differences in treatment outcomes between central and peripheral ES‐SCLC.

## 3 DISCUSSION

**FIGURE 8:**
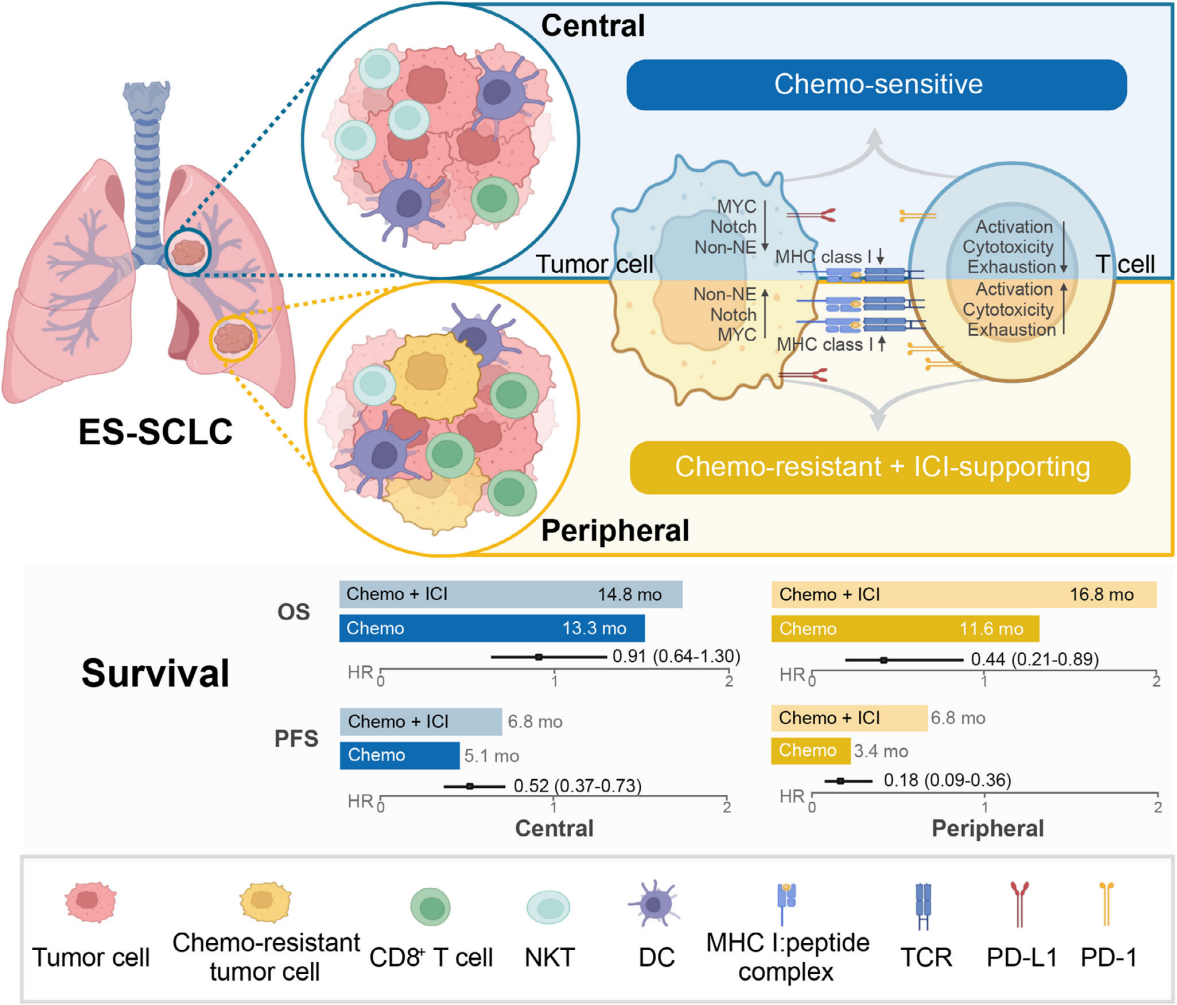
Schematic diagram of the study. ES‐SCLC, extensive‐stage small‐cell lung cancer.

In this study, we first compared the treatment responses of central and peripheral ES‐SCLC to front‐line therapy and explored the mechanisms behind their differing outcomes (Figure 8). Results indicated that the peripheral type had a significantly lower ORR and shorter PFS with chemotherapy alone but showed greater benefit from chemo‐immunotherapy compared with the central type. This suggests that peripheral ES‐SCLC may possess chemo‐resistant and ICI‐supportive characteristics. Mechanistically, scRNA‐seq analysis revealed upregulation of the chemo‐resistance‐related MYC‐Notch‐non‐NE axis and a more immune‐responsive tumor microenvironment in peripheral ES‐SCLC. Public data from the IMpower133 trial further validated the chemo‐resistant role of the MYC‐Notch‐non‐NE axis and highlighted the importance of the inhibitory Notch ligand, DLL3, in predicting response to chemo‐immunotherapy and promoting an immune‐supportive tumor phenotype.

Previous studies indicate that central and peripheral SCLC are distinct entities with differences in clinicopathological and genomic features, as well as prognosis.^16^, ^18^, ^19^, ^20^, ^21^, ^22^ However, the responses of these two types of ES‐SCLC to front‐line treatments are not well understood. In our clinical cohort of 265 ES‐SCLC patients, we observed that within the chemotherapy group, peripheral ES‐SCLC had a significantly lower ORR (44.8% vs. 71.2%) and shorter PFS (3.4 vs. 5.1 months) compared with the central type, which aligns with findings from other studies.^16^, ^22^ Interestingly, Nobuhiro et al.^18^ reported that peripheral SCLC was associated with longer OS, likely due to their cohort having a high proportion of peripheral LS‐SCLC patients (46.2%). Additionally, when comparing chemo‐immunotherapy with chemotherapy, peripheral‐type patients demonstrated a more substantial reduction in the risk of disease progression and mortality than central‐type patients. These results suggest that central and peripheral ES‐SCLC exhibit distinct responses to front‐line therapy. Similarly, in RAS wild‐type metastatic colorectal cancer, patients with left‐sided tumors experienced significantly improved OS when panitumumab, rather than bevacizumab, was added to standard front‐line chemotherapy.^50^ Such findings highlight the potential of primary tumor location as a predictive biomarker for treatment efficacy in ES‐SCLC.

Recently, scRNA‐seq analyses showed the potential to comprehensively understand the biology of SCLC. Chan et al.^37^ adopted scRNA‐seq to investigate the heterogeneity and tumor microenvironment of SCLC and identified a PLCG2‐high‐expressing subpopulation associated with metastasis and poor prognosis. However, this cohort included patients across various clinical stages (IA‐IV), treatment statuses (mostly postchemotherapy), and sampling sites (lung and multiple metastatic sites) and was predominantly Caucasian. In the setting of LS‐SCLC, Cheng et al.^36^ conducted comparative analyses of treatment‐naïve and posttreatment LS‐SCLC specimens and found that chemotherapy would tremendously remodel SCLC and their TME. Similarly, Tian et al.^35^ have included 11 LS‐SCLC patients for scRNA‐seq analyses and decoded substantial intratumor heterogeneity (ITH) and an inflamed phenotype in non‐NE tumors. In our study, all the patients enrolled were treatment‐naïve ES‐SCLC and we observed similar coarse cell types and substantial heterogeneity among patients. Nevertheless, we noticed significantly fewer nonmalignant cell infiltrates in ES‐SCLC (10.9% in our study) compared with LS‐SCLC (36.5% and 64.9% of untreated and treated tumor tissues from Cheng's, and 46.3% from Tian's). In addition, we observed pronounced co‐expression of subtype‐specific TFs within single samples. These discrepancies may stem from tumor heterogeneity and the exceptional plasticity of SCLC.

Importantly, we demonstrated the distinct tumoral and microenvironmental characteristics of central and peripheral ES‐SCLC. Compared with the central type, tumor cells from peripheral tumors seemed more chemo‐rejective and ICI‐supportive. T cells from peripheral tumors highly expressed anti‐tumor immunity and exhaustion‐related genes, which could be exploited by PD1/PD‐L1 blockade to enhance therapeutic effects. Previously, central and peripheral lung tumors were implicated in discrete histological types, growth patterns, mutational landscapes, metastatic statuses, and survival prognosis.^51^, ^52^ With regard to SCLC, Sutherland et al.^53^ found that SPC‐positive mouse alveolar type 2 cells could be transformed into SCLC following loss of *Trp53* and *Rb1*, and showed a strong predilection for peripheral location, whereas tumors induced from NE cells were primarily centrally located. Eisaku et al.^22^ also demonstrated that peripheral SCLC accounted for the majority of TTF1 (a vital marker of lung adenocarcinoma) immunoreactive tumors. Furthermore, Wang et al.^21^ uncovered the distinct genomic features of central and peripheral SCLC. Similarly, we found that a substantial proportion of peripheral tumor cells highly expressed non‐NE genes, while central tumor cells mostly expressed NE genes. Taken together, these findings suggested that central and peripheral ES‐SCLC have different biological characteristics that lead to distinct responses to anticancer therapy.

Mechanistically, differences in MYC‐Notch‐non‐NE axis activation between central and peripheral ES‐SCLC may explain their distinct treatment responses. In peripheral ES‐SCLC, this axis was highly activated. In a study by Ireland et al.,^40^ MYC reprogrammed SCLC non‐NE differentiation by activating Notch signaling. Our findings further connected this axis to chemo‐resistance in ES‐SCLC. External data from IMpower133 revealed that activation of this axis was strongly predictive of poorer outcomes with chemotherapy alone. Furthermore, a recent study by Kim et al.^47^ highlighted the predictive significance of NOTCH1 expression for OS in patients treated with atezolizumab and chemotherapy. In our study, we similarly found that the inhibitory Notch ligand, DLL3, was downregulated in peripheral ES‐SCLC, and its expression level served as a critical predictor in chemo‐immunotherapy. Specifically, DLL3 expression was negatively associated with OS in patients receiving chemo‐immunotherapy. Additionally, patients with low DLL3 expression had significantly prolonged OS with chemo‐immunotherapy compared with chemotherapy alone, while those with elevated DLL3 expression did not. These findings underscore the essential role of Notch signaling in ES‐SCLC's response to anti‐tumor regimens.

This study has several limitations. First, although we reviewed patients over an extended period, only 265 patients were ultimately included in the clinical cohort. In comparisons of outcomes between central and peripheral ES‐SCLC receiving chemo‐immunotherapy, as well as between chemo‐immunotherapy and chemotherapy groups within the central type, sample sizes were relatively limited, warranting further studies with larger cohorts. Second, due to the relatively small sample size and the use of biopsy specimens for scRNA‐seq, our findings primarily highlight distinct tumor and microenvironmental characteristics associated with different primary tumor locations. Third, our mechanistic analyses suggested that differential activation of the MYC‐Notch‐non‐NE axis may contribute to the varied responses of the two ES‐SCLC types to front‐line therapies. However, further validation through cellular and animal studies is necessary.

In conclusion, our study revealed that central and peripheral ES‐SCLC exhibit distinct responses to front‐line chemotherapy and chemo‐immunotherapy. Differences in the activation of the MYC‐Notch‐non‐NE axis may offer a mechanistic explanation for these variations. Additionally, our findings underscore the predictive value of DLL3 expression in determining response to chemo‐immunotherapy in ES‐SCLC.

## 4 MATERIALS AND METHODS

### 4.1 Patient cohort for outcome comparison

Patients diagnosed with SCLC at Shanghai Pulmonary Hospital from May 2015 to Jan 2022 were retrospectively reviewed. Patients who met the following criteria were enrolled in this study: (1) Cytological/histological diagnosis of SCLC, staged as ES‐SCLC according to VALG at the baseline of front‐line therapy. ES‐SCLC is defined as disease involvement that has spread beyond a single radiation port.^54^ (2) Front‐line treatment was either chemotherapy or chemo‐immunotherapy, regardless of the specific chemotherapy or immunotherapy drugs used. (3) Primary tumor location could be classified as central or peripheral. Specifically, two experienced radiologists independently assessed the patients’ chest CT images. The assessments were categorized into three levels: central (C), peripheral (P), and indistinguishable (X). Representative CT images of central and peripheral ES‐SCLC are shown in Figures S7A,B. Indistinguishable cases often result from significant pleural effusion, obstructive pneumonia, obstructive atelectasis, or multiple lesions making the primary site unclear. If the two radiologists reached a consensus, results CC, PP, or XX were classified as central, peripheral, or indistinguishable, respectively. If there was no consensus, leading to results CP, CX, or PX, a third radiologist would be consulted for judgment. The final classification was based on the determination of the three radiologists: CCP and CCX were determined as central, PPC and PPX were judged as peripheral, and other results were decided as indistinguishable (Figure S8). (4) Complete clinical data, efficacy and prognosis details of the front‐line treatment were available.

Of 1522 SCLC patients screened, 265 met the inclusion criteria and were enrolled. Clinical data and treatment details were collected, including age, gender, smoking status, comorbidities, Eastern Cooperative Oncology Group performance status (ECOG PS), treatment strategies, and outcomes of the front‐line treatment.

### 4.2 Efficacy evaluation

Tumor responses were assessed according to response evaluation criteria in solid tumors (RECIST, version 1.1). PFS was defined as the time interval from the date of treatment initiation to the date of disease progression or death of any cause, whichever occurred first. Patients without disease progression or death were censored. OS was defined as the date of treatment initiation to the date of death of any cause or last follow‐up in surviving patients.

### 4.3 scRNA sequencing and analysis

Patients admitted to Shanghai Pulmonary Hospital from January to March 2023 were monitored and those meeting the following criteria were enrolled for scRNA‐seq: (1) diagnosed with SCLC by cytology or histology; (2) classified with ES‐SCLC by Veterans Administration Lung Cancer Study Group; (3) had not received systemic anti‐tumor treatment; (4) were distinguishable as central or peripheral type; (5) provided written informed consent. ES‐SCLC is defined as disease involvement that has spread beyond a single radiation port.^54^ A total of nine patients were enrolled and the specimens were collected from primary lung tumor by diagnostic procedures including transcutaneous needle biopsy or bronchoscopy. The clinicopathological characteristics of the nine patients were recorded. Among them, five patients had central ES‐SCLC, while four had peripheral ES‐SCLC.

#### 4.3.1 Sample preparation and scRNA‐seq

The process of tissue dissociation, single‐cell suspension preparation, single‐cell RNA‐sequencing library preparation, and generation of single‐cell gene expression matrices were detailly described in our previous work.^29^

#### 4.3.2 Quality control, cell type clustering, and cell type identification

In the process of quality control, cells expressing either less than 200 or more than 5000 genes were removed. Those with more than 20,000 UMIs or expressing more than 30% mitochondrial genes were also discharged. Ultimately, 12,897 cells were preserved for subsequent analyses. R package Seurat 4.3^55^ was mainly used in the downstream data analyzing process. Functions NormalizeData and ScaleData were used to normalize expression matrices. Then, the FindVariableFeatures function was used to determine the top 1000 most variable genes, which were further subjected to principal component analysis (PCA) with the use of the function RunPCA. The top nine principal components and a resolution of 0.6 were used with the function FindClusters to generate 16 clusters. The major steps of dimensionality reduction and clustering are shown in Figure S9. Through the expression of canonical markers (Table S6), cells were divided into seven major cell types: epithelial cells (CAPS, SNTN), alveolar cells (CLDN18, AQP4, FLOR1), mesenchymal cells (COL1A1, COL1A2, DCN), T cells (CD2, CD3D, CD3E, CD3G), B cells (CD79A, CD79B), myeloid cells (CD14, LYZ), and cancer cells. The cancer cell cluster was negative for normal lung epithelial markers and positive for EPCAM and further verified by InferCNV. These results were visualized by uniform manifold approximation and projection (UMAP). We further clustered T‐, B‐, myeloid‐, and mesenchymal cells. T cells were subdivided into NKT (NKG7) and CD8^+^ T cells (CD8A); B cells were clustered into follicular B cells (MS4A1, MHC‐II, CXCR4) and plasma cells (MZB1, JCHAIN, IGH); myeloid cells were separated into monocytes (CD14, LYZ, VCAN), SLC40A^+^ macrophages (CD68, SLC40A), and CCL3/4^+^ macrophages (CD68, CCL3, CCL4); while mesenchymal cells were further split into fibroblasts (COL1A1) and endothelial cells (CLDN5).

#### 4.3.3 Differential expression analysis

The DEGs were calculated by function FindMarkers in the R package Seurat with a log foldchange threshold of 0.5 and a minimum different fraction of 0.1.

#### 4.3.4 Pathway enrichment analysis

Enriched pathways of DEGs were identified by the R package clusterProfiler,^56^ and the top 15 enriched GO:BP pathways were shown. To assess different pathway activities for each cell, we implemented GSVA with Hallmark gene sets downloaded from MSigDB (https://www.gsea‐msigdb.org/gsea/msigdb). For T‐ and B cells, we used the GO biological process gene sets. R package limma^57^ was used to identify the differential pathway activity between central and peripheral type cells. The Benjamini‐Hochberg method was applied for *p*‐value adjustment to control the false discovery rate. Pathways with an adjusted *p*‐value below 0.05 were visualized using bar plots, displaying the *t*‐values of the GSVA scores.

#### 4.3.5 Cell activity scores

We used the function AddModuleScore implemented in Seurat to evaluate cell activity between central and peripheral type cells. For T cells, the gene list was downloaded from gene ontology (http://geneontology.org/) with an accession of GO:0042110. For cancer cells, we input gene lists of the hallmark Notch signaling pathway, hallmark WNT beta‐catenin signaling pathway, and 50 NE‐associated genes from Zhang's research.^44^

#### 4.3.6 CNA detection

The CNA of each patient was inferred using the R package InferCNV.^58^ Immune cells (T‐ and B cells) were used as baselines to estimate the CNA of malignant cells. Copy number information was estimated by sorting the analyzed genes by their chromosomal location and applying a moving average to the relative expression values, with a sliding window within each chromosome. We used the default parameters to run InferCNV. The CNV scores were obtained by subtracting 1 from the output matrix and taking the absolute values. Two‐sample Wilcoxon test was used to test the difference in means of CNV scores.

#### 4.3.7 Intercellular interaction analysis

CellChat^59^ was used to analyze intercellular communication networks from the scRNA‐seq data. We followed the default settings of the R package CellChat. Based on the list of significant interactions, we assessed the enrichment of interactions using Fisher's exact test and illustrated the LR pairs significantly enriched in both central and peripheral types.

### 4.4 Analysis of clinical and transcriptomic data from IMpower133

The phenotype and transcriptomic data from IMpower133 were downloaded from the European Genome‐phenome Archive (EGAS00001004888). GSVA was conducted to assess pathway activities of Notch signaling, MYC_TARGETS_V1, MYC_TARGETS_V2, MYC_UP_V1_UP, as well as NE and non‐NE differentiation. The gene sets for the first four pathways were sourced from MSigDB, while the gene sets for NE and non‐NE differentiation were obtained from a prior study.^44^ Median values of gene log2(TPM+1) expression data or pathway GSVA scores were used as cut‐off points to differentiate between low and high‐expression groups. The difference in gene expression between low and high DLL3 expression groups was assessed using batch rank sum tests on all genes. The results of the differential expression analysis were applied to perform GSEA with hallmark gene sets from MSigDB.

### 4.5 Statistical analysis

The categorical variables were compared using the chi‐square test or Fisher's exact test when needed. PFS and OS were estimated by the Kaplan–Meier method and compared the significance of difference by log‐rank test. The Cox proportional hazard model was performed for univariate and multivariate survival analyses to calculate the hazard ratio (HR) and 95% confidence interval (CI). Parameters with *p*‐value of univariate less than 0.1 were included in the multivariate model. *p*‐values were two‐sided and considered statistically significant differences when less than 0.05. All the statistical analyses were performed using the SPSS statistical software (version 22.0; IBM Corporation). Plots were created with R software (version 4.2.2).

## AUTHOR CONTRIBUTIONS

Fengying Wu, Shengxiang Ren, Luonan Chen, and Caicun Zhou designed this study and revised the manuscript. Libo Luo, Rui Xia, and Shiqi Mao drafted the manuscript. Libo Luo, Rui Xia, Shiqi Mao, Qian Liu, He Du, Tao Jiang, Shuo Yang, Yan Wang, Wei Li, Fei Zhou, Jia Yu, and Guanghui Gao collected and assembled the data. Libo Luo, Rui Xia, Shiqi Mao, Qian Liu, He Du, Xuefei Li, Chao Zhao, Lei Cheng, Jingyun Shi, and Xiaoxia Chen performed data analysis and interpretation. All authors were involved in manuscript writing. All authors have read and approved the final manuscript.

## CONFLICT OF INTEREST STATEMENT

The authors declare no conflict of interest.

## ETHICS STATEMENT

The study was approved by the Shanghai Pulmonary Hospital Ethics Committee (no. K24493). Written informed consent was obtained from the participants.

## Supporting information



Supporting Information

Supporting Information

## Data Availability

scRNA‐seq data of this paper have been deposited in the OMIX, China National Center for Bioinformation Beijing Institute of Genomics, Chinese Academy of Sciences (accession number: OMIX006823). Other data supporting the findings of this study are available from the corresponding author upon reasonable request.
