# Supplementary material for: Differential expression of the MYC‐Notch axis drives divergent responses to the front‐line therapy in central and peripheral extensive‐stage small‐cell lung cancer
Source: MedComm (2020). 2025 Feb 18;6(3):e70112. doi: 10.1002/mco2.70112 (PMC11836348; doi:10.1002/mco2.70112)
Supplement: Supplementary file 1 — Supporting Information [file MCO2-6-e70112-s002.docx]

**Distinct single-cell transcriptomic characteristics and responses to the front-line therapy in patients with central or peripheral extensive-stage small-cell lung cancer**

Libo Luo, Rui Xia, Shiqi Mao, Qian Liu, He Du, Tao Jiang, Shuo Yang, Yan Wang, Wei Li, Fei Zhou, Jia Yu, Guanghui Gao, Xuefei Li, Chao Zhao, Lei Cheng, Jingyun Shi, Xiaoxia Chen, Caicun Zhou, Luonan Chen, Shengxiang Ren, Fengying Wu

**Figure S1……………………………..…………………………………………………………….2**

**Figure S2……………………………..…………………………………………………………….3**

**Figure S3……………………………..…………………………………………………………….4**

**Figure S4……………………………..…………………………………………………………….5**

**Figure S5……………………………..…………………………………………………………….6**

**Figure S6……………………………..…………………………………………………………….7**

**Figure S7……………………………..…………………………………………………………….8**

**Figure S8……………………………..…………………………………………………………….9**

**Figure S9……………………………..…………………………………………………………….10**

**Table S1.……………………………..……………………………………………………………..11**

**Table S2.……………………………..……………………………………………………………..12**

**Table S3.……………………………..……………………………………………………………..13**

**Table S4.……………………………..……………………………………………………………..14**

**Table S5.……………………………..……………………………………………………………..18**

**
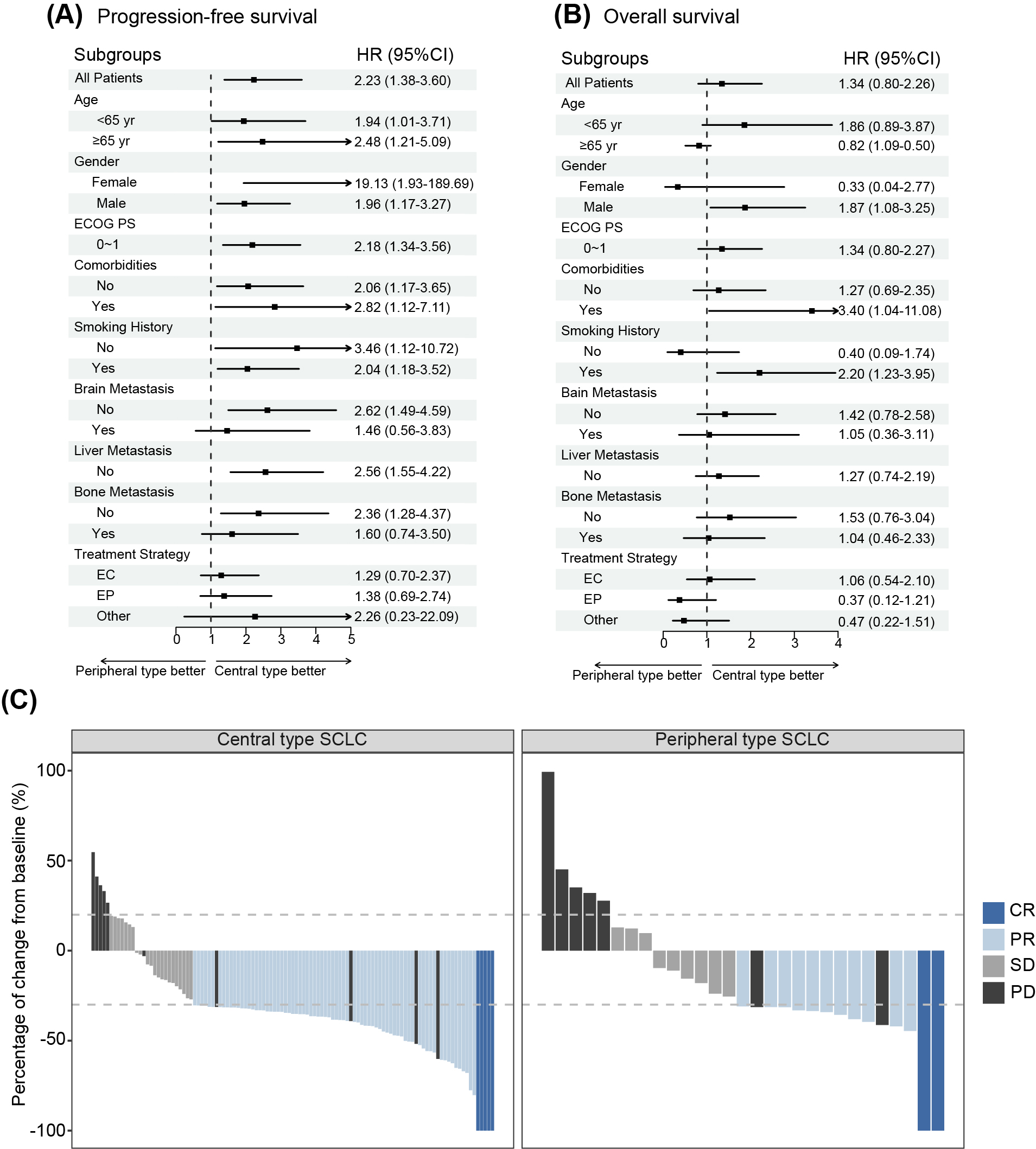
**

**Figure S1** Forest plots of subgroup analysis for (A) progression-free survival or (B) overall survival; and (C) best percentage change from baseline in the size of targeted lesions in patients treated with chemotherapy. ECOG PS, Eastern Corporation Oncology Group performance status. EC, Etoposide & Carboplatin; EP, Etoposide & Cisplatin. SCLC, Small-cell lung cancer. CR, Complete response; PR, Partial response; SD, Stable disease; PD, Progressive disease.
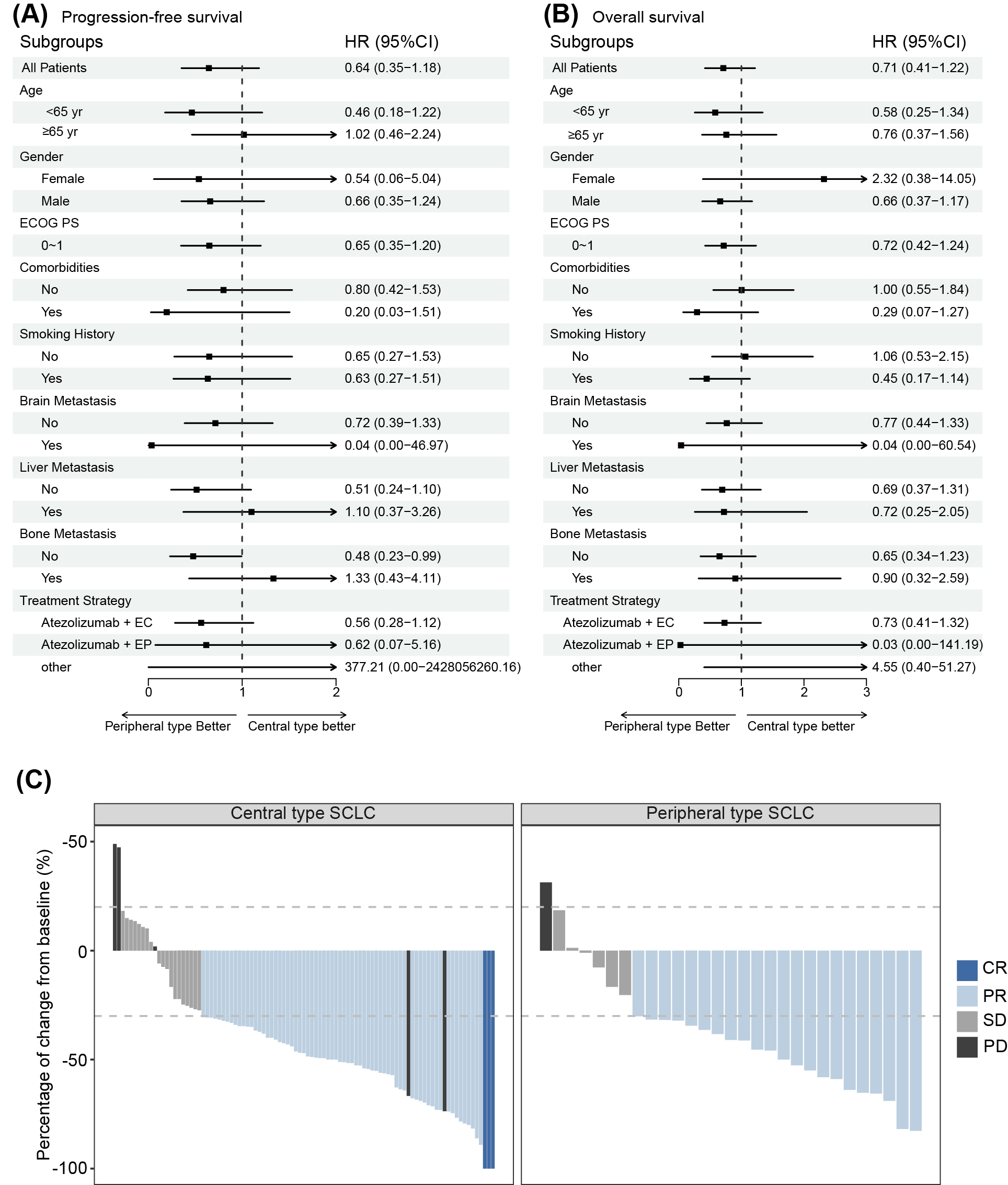


**Figure S2** Forest plots of subgroup analysis for (A) progression-free survival or (B) overall survival; and (C) best percentage change from baseline in the size of targeted lesions in patients treated with chemo-immunotherapy. ECOG PS, Eastern Corporation Oncology Group performance status. EC, Etoposide & Carboplatin; EP, Etoposide & Cisplatin. SCLC, Small-cell lung cancer. CR, Complete response; PR, Partial response; SD, Stable disease; PD, Progressive disease.

**
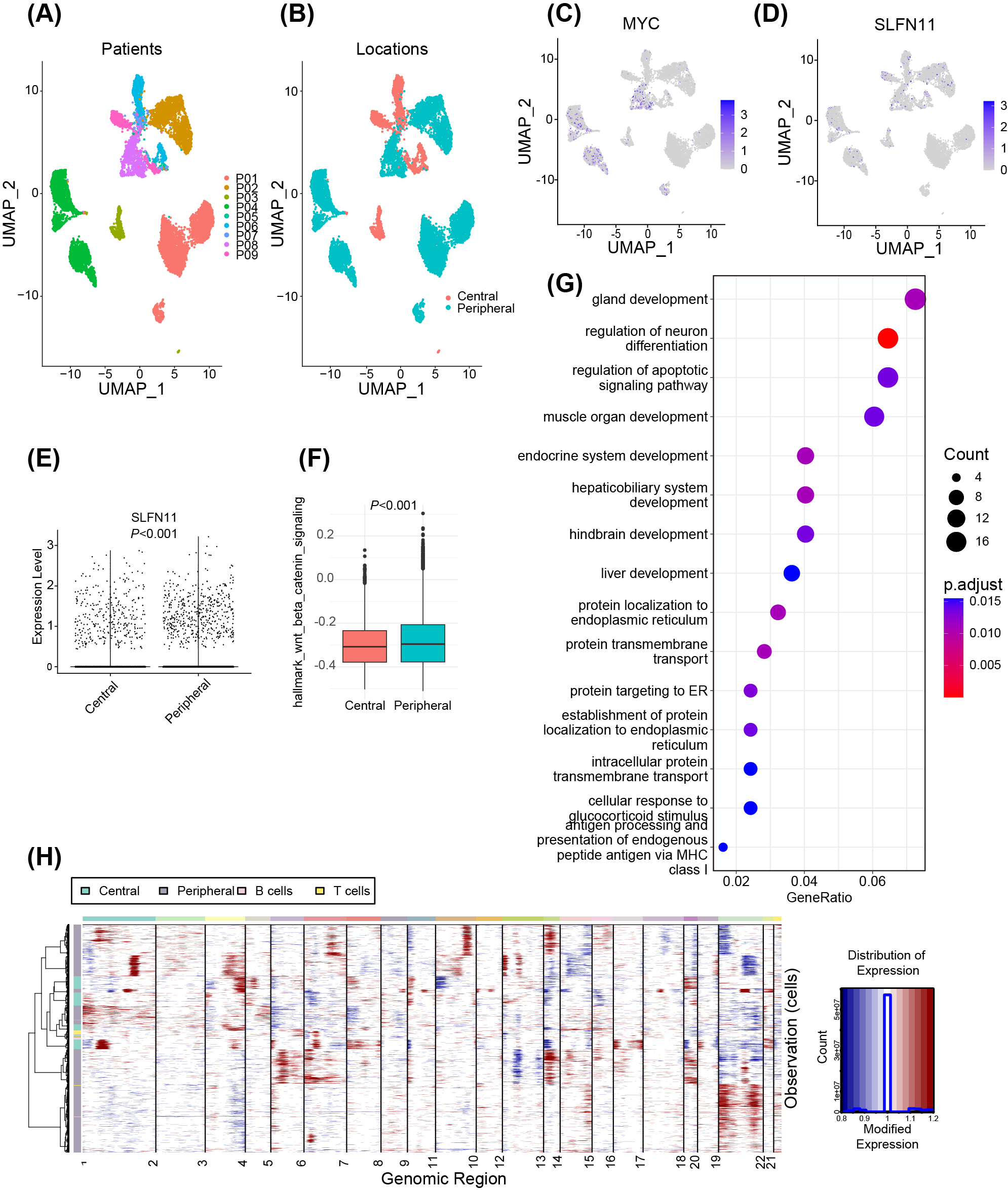
**

**Figure S3** UMAP plots of 11,718 tumor cells, colored by (A) patients or (B) primary tumor locations; UMAP plots depicting expression levels of (C) MYC or (D) SLFN11 in tumor cells; (E) Expression level of SLFN11 in individual tumor cell; (F) Cell activity score of Wnt/β-catenin signaling for each cancer cell; (G) Top 15 enriched pathways from GO enrichment analysis of differentially expressed genes between central and peripheral cancer cells; (H) Heatmap of CNA profiles inferred from scRNA-seq of cancer cells. In (E)-(G), (n=9,594 cells for peripheral type and n=2,124 cells for central type). UMAP, Uniform manifold approximation and projection. GO, Gene ontology. CNA, Copy number alteration.

**
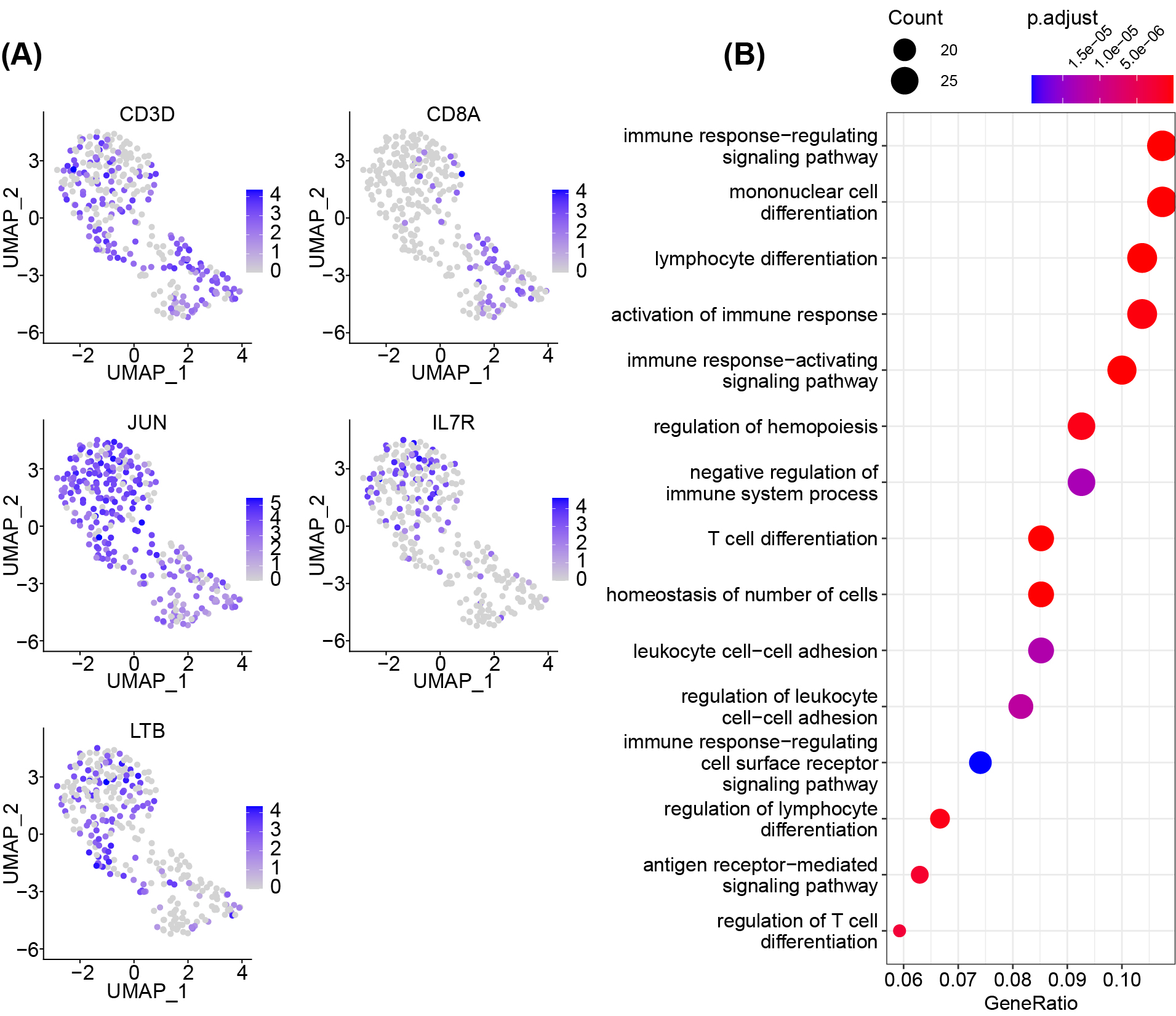
**

**Figure S4** (A) Expression of marker genes of T cell subclusters; (B) Top 15 enriched pathways from GO enrichment analysis of differentially expressed genes between central and peripheral T cells (n=114 T cells for peripheral type and n=185 T cells for central type). UMAP, Uniform manifold approximation and projection. GO, Gene ontology.

**
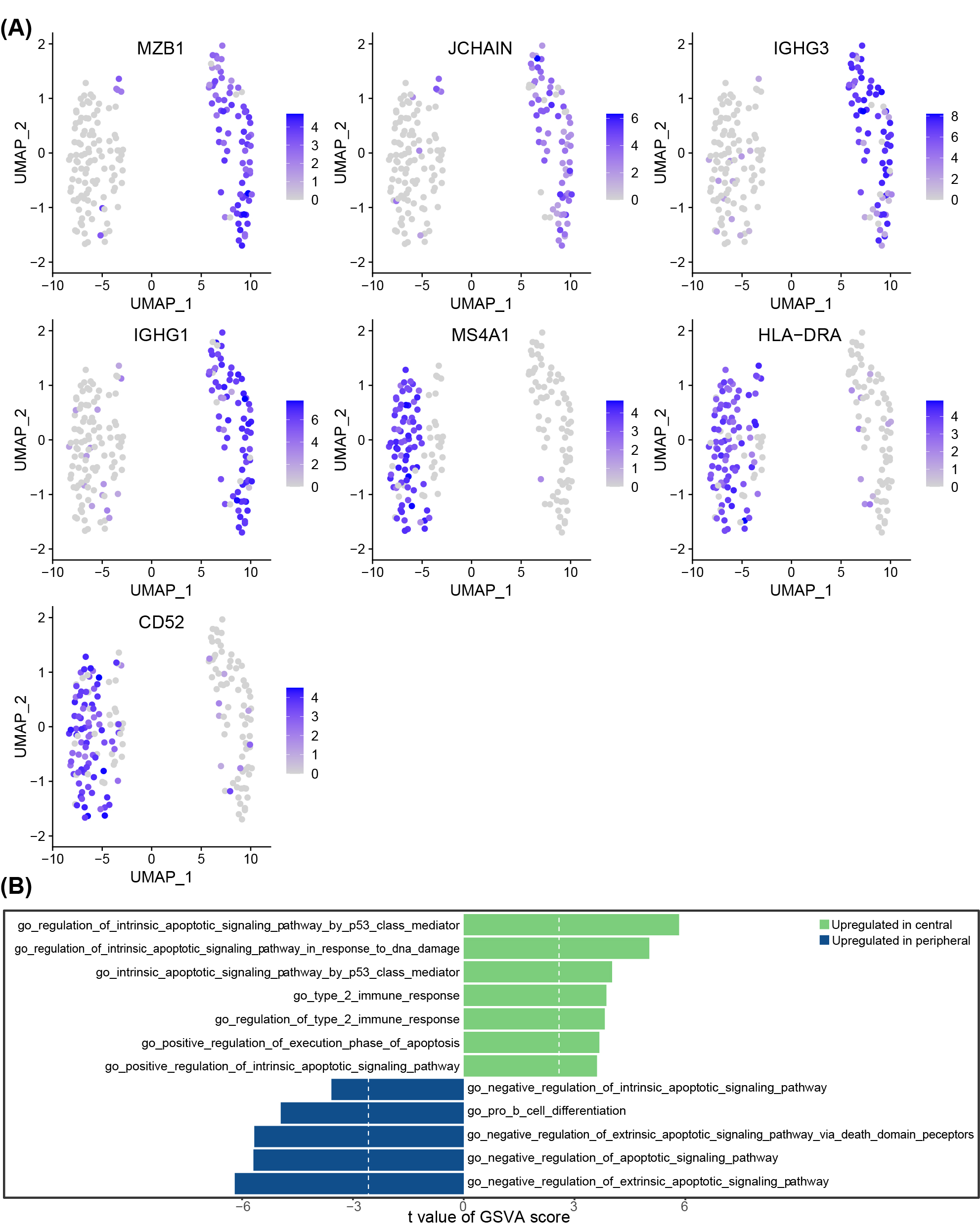
**

**Figure S5** (A) Expression of marker genes of B cell subclusters; (B) Differences in Hallmark pathway activities scored per cell by GSVA between central and peripheral B cells, the vertical white dotted lines denote critical T values with adjusted *P* values of 0.05 (n=66 B cells for peripheral type and n=121 B cells for central type). UMAP, Uniform manifold approximation and projection. GSVA, Gene set variation analysis.

**
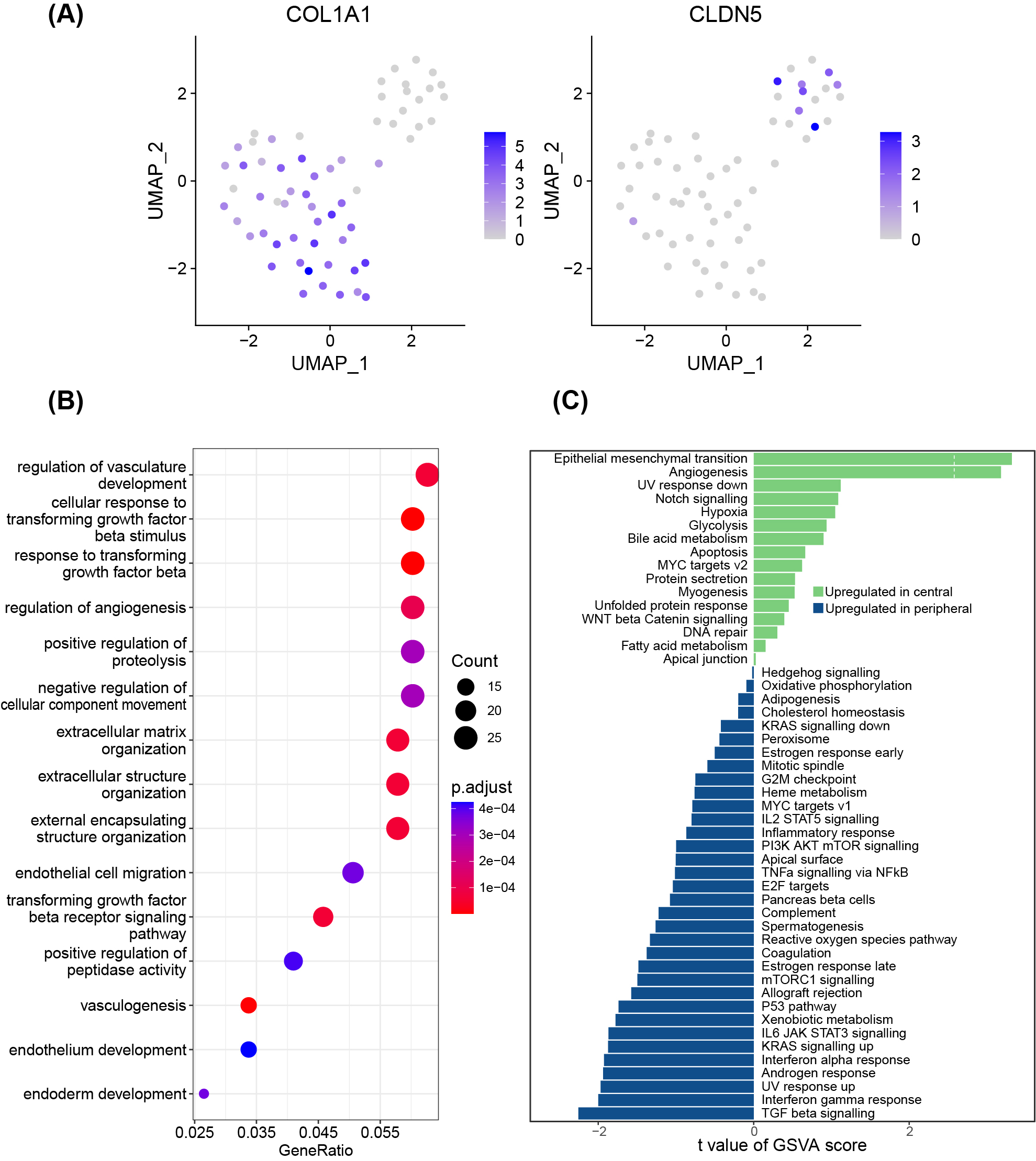
**

**Figure S6** (A) Expression of marker genes of Mesenchymal cell subclusters; (B) Top 15 enriched pathways from GO enrichment analysis of differentially expressed genes between central and peripheral Mesenchymal cells; (C) Differences in Hallmark pathway activities scored per cell by GSVA between central and peripheral Mesenchymal cells, the vertical white dotted lines denote critical T values with adjusted *P* values of 0.05. In (B) and (C), n=43 Mesenchymal cells for peripheral type and n=19 Mesenchymal cells for central type. UMAP, Uniform manifold approximation and projection. GO, Gene ontology. GSVA, Gene set variation analysis.


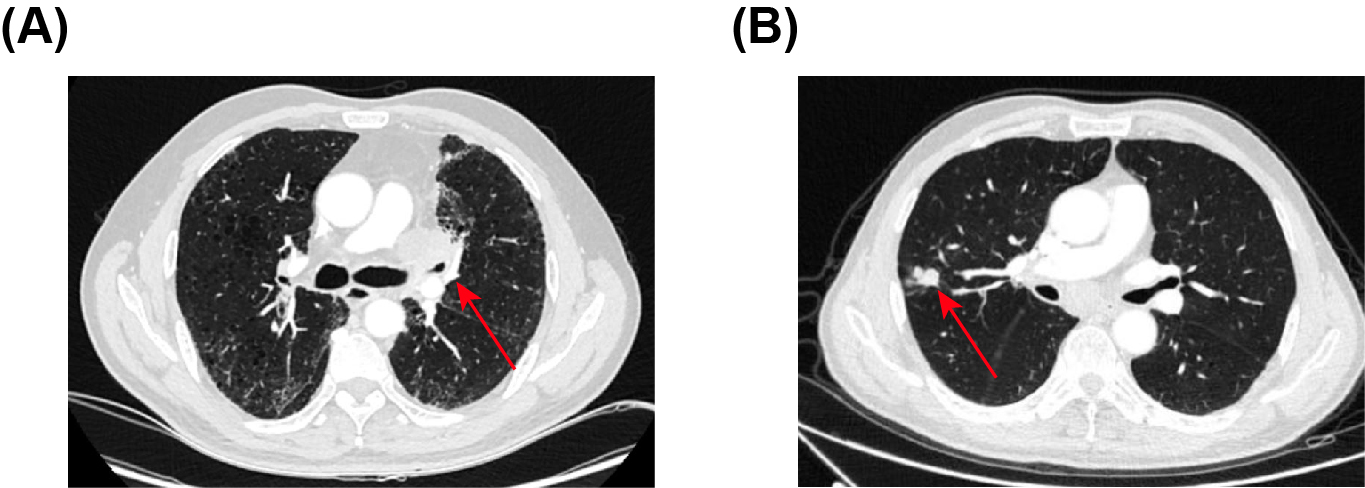


**Figure S7** Representative CT images of (A) central and (B) peripheral ES-SCLC. The locations of the lesions are indicated by the red arrows.

**
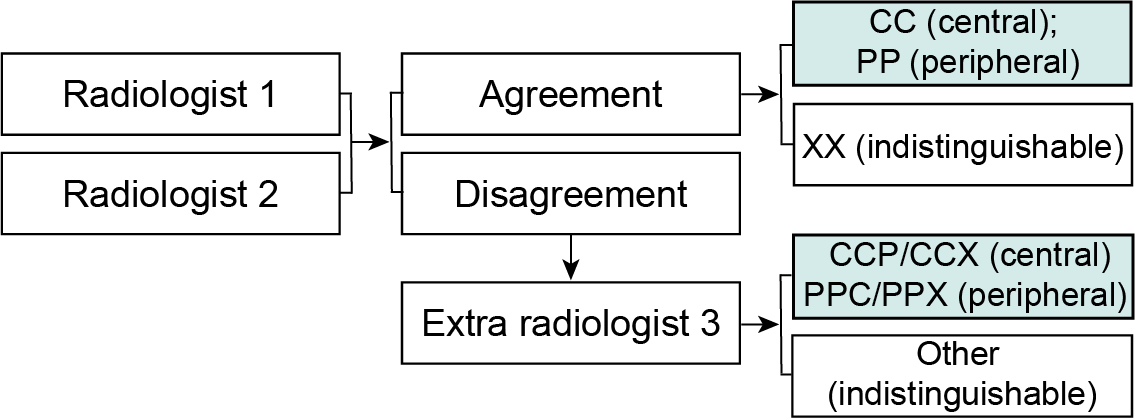
**

**Figure S8** Flow chart showing the process to determine the primary tumor location.


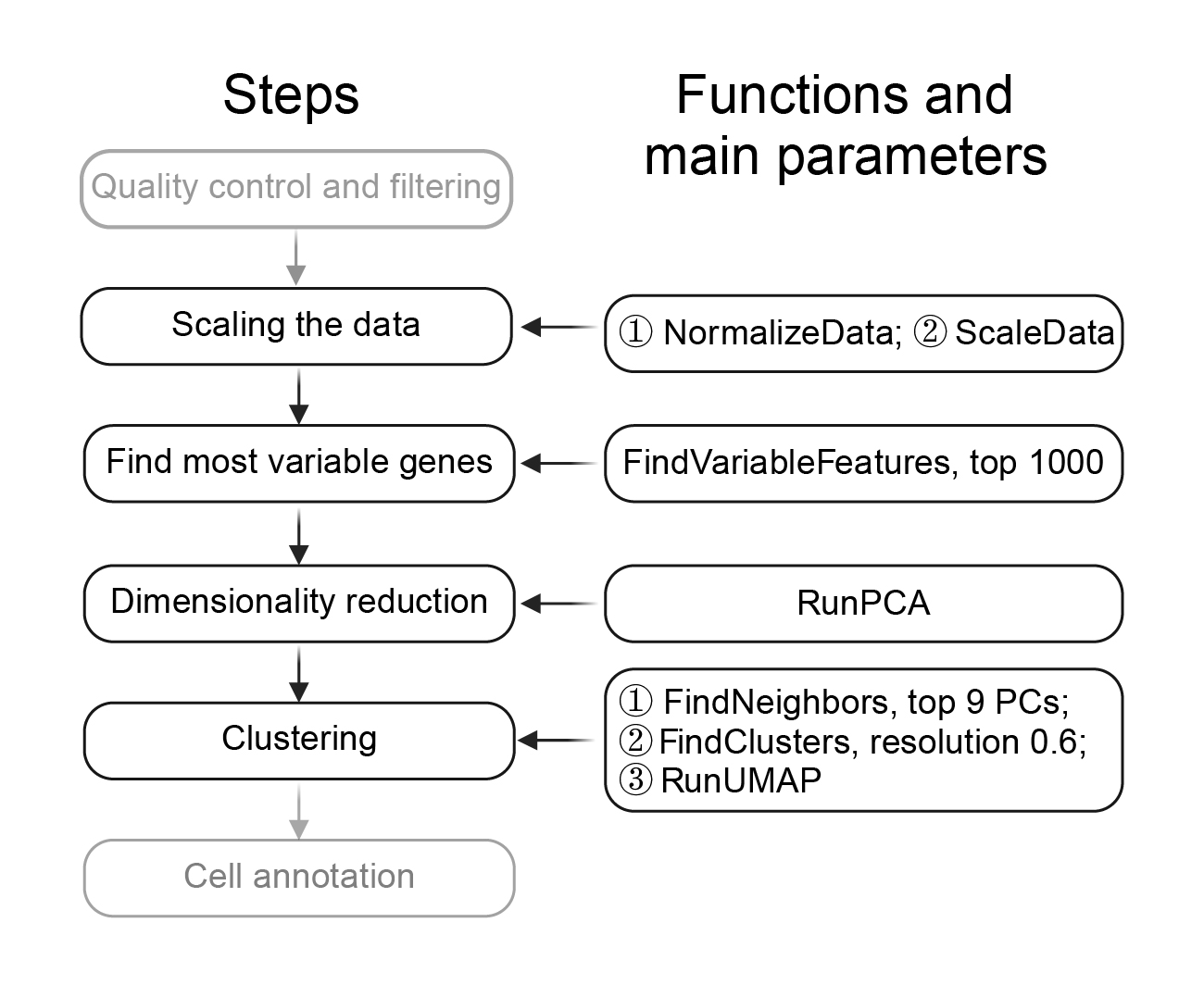


**Figure S9** Major steps of dimensionality reduction and clustering.

**Table S1.** Baseline characteristics of patients in this study.

| **Item** | **Total**  **(n=265)** | **Chemotherapy (n=140)** | | |  | **Chemo-immunotherapy (n=125)** | | |  | **Central (n=207)** | | |  | **Peripheral (n=58)** | | |
| --- | --- | --- | --- | --- | --- | --- | --- | --- | --- | --- | --- | --- | --- | --- | --- | --- |
|  |  | **Central**  **(n=111)** | **Peripheral**  **(n=29)** | ***P***  **value** |  | **Central**  **(n=96)** | **Peripheral**  **(n=29)** | ***P***  **value** |  | **Chemotherapy(n=111)** | **Chemo-immunotherapy**  **(n=96)** | ***P***  **value** |  | **Chemotherapy(n=29)** | **Chemo-immunotherapy**  **(n=29)** | ***P***  **value** |
| **Age, yr, No (%)** |  |  |  |  |  |  |  |  |  |  |  |  |  |  |  |  |
| Median (range) | 65 (59, 69) | 64 (41, 81) | 65 (55, 73) |  |  | 65 (59, 70) | 65 (62, 70) |  |  | 64 (41, 81) | 65 (59, 70) |  |  | 65 (55, 73) | 65 (62, 70) |  |
| ≥65 years | 133 (50.2) | 53 (47.7) | 15 (51.7) | 0.763 |  | 50 (52.1) | 15 (51.7) | 0.973 |  | 53 (47.7) | 50 (52.1) | 0.534 |  | 15 (51.7) | 15 (51.7) | 1.000 |
| <65 years | 132 (49.8) | 58 (52.3) | 14 (48.3) |  |  | 46 (47.9) | 14 (48.3) |  |  | 58 (52.3) | 46 (47.9) |  |  | 14 (48.3) | 14 (48.3) |  |
| **Gender, No (%)** |  |  |  |  |  |  |  |  |  |  |  |  |  |  |  |  |
| Male | 241 (90.9) | 99 (89.2) | 26 (89.7) | 1.000 |  | 89 (92.7) | 27 (93.1) | 1.000 |  | 99 (89.2) | 89 (92.7) | 0.382 |  | 26 (89.7) | 27 (93.1) | 1.000 |
| Female | 24 (9.1) | 12 (10.8) | 3 (10.3) |  |  | 7 (7.3) | 2 (6.9) |  |  | 12 (10.8) | 7 (7.3) |  |  | 3 (10.3) | 2 (6.9) |  |
| **ECOG PS, No (%)** |  |  |  |  |  |  |  |  |  |  |  |  |  |  |  |  |
| 0~1 | 260 (98.1) | 110 (99.1) | 28 (96.6) | 0.373 |  | 93 (96.9) | 29 (100.0) | 1.000 |  | 110 (99.1) | 93 (96.9) | 0.514 |  | 28 (96.6) | 29 (100.0) | 1.000 |
| 2 | 5 (1.9) | 1 (0.9) | 1 (3.4) |  |  | 3 (3.1) | 0 (0.0) |  |  | 1 (0.9) | 3 (3.1) |  |  | 1 (3.4) | 0 (0.0) |  |
| **Smoking history, No (%)** |  |  |  |  |  |  |  |  |  |  |  |  |  |  |  |  |
| Never smoker | 87 (32.8) | 27 (24.3) | 6 (20.7) | 0.681 |  | 38 (39.6) | 16 (55.2) | 0.137 |  | 27 (24.3) | 38 (39.6) | 0.018 |  | 6 (20.7) | 16 (55.2) | 0.007 |
| Former/current smoker | 178 (67.2) | 84 (75.7) | 23 (79.3) |  |  | 58 (60.4) | 13 (44.8) |  |  | 84 (75.7) | 58 (60.4) |  |  | 23 (79.3) | 13 (44.8) |  |
| **Comorbidities, No (%)** |  |  |  |  |  |  |  |  |  |  |  |  |  |  |  |  |
| No | 173 (65.3) | 71 (64.0) | 19 (65.5) | 0.876 |  | 60 (62.5) | 23 (79.3) | 0.093 |  | 71 (64.0) | 60 (62.5) | 0.828 |  | 19 (65.5) | 23 (79.3) | 0.240 |
| Yes | 92 (34.7) | 40 (36.0) | 10 (34.5) |  |  | 36 (37.5) | 6 (20.7) |  |  | 40 (36.0) | 36 (37.5) |  |  | 10 (34.5) | 6 (20.7) |  |
| **Metastatic sites, No (%)** |  |  |  |  |  |  |  |  |  |  |  |  |  |  |  |  |
| Brain | 57 (21.5) | 33 (29.7) | 6 (20.7) | 0.382 |  | 16 (16.7) | 2 (6.9) | 0.312 |  | 33 (29.7) | 16 (16.7) | 0.027 |  | 6 (20.7) | 2 (6.9) | 0.231 |
| Liver | 51 (19.2) | 20 (18.0) | 1 (3.4) | 0.076 |  | 22 (22.9) | 8 (27.6) | 0.606 |  | 20 (18.0) | 22 (22.9) | 0.382 |  | 1 (3.4) | 8 (27.6) | 0.030 |
| Bone | 79 (29.8) | 34 (30.6) | 13 (44.8) | 0.149 |  | 25 (26.0) | 7 (24.1) | 0.837 |  | 34 (30.6) | 25 (26.0) | 0.466 |  | 13 (44.8) | 7 (24.1) | 0.097 |

**Abbreviations:** ECOG PS, Eastern Corporation Oncology Group performance status.

**Table S2.** Treatment information.

| **Group** | **Treatment strategy,**  **No (%)** | **Total** | **Central-type** | **Peripheral-type** | ***P* value** |
| --- | --- | --- | --- | --- | --- |
| **The chemotherapy group** |  |  |  |  |  |
|  | EP | 66 (47.1) | 53 (47.8) | 13 (44.8) | 0.103 |
|  | EC | 63 (45.0) | 52 (46.8) | 11 (37.9) |  |
|  | Other | 11 (7.9) | 6 (5.4) | 5 (17.3) |  |
|  | Sum | 140 (100.0) | 111 (100.0) | 29 (100.0) |  |
| **The chemo-immunotherapy**  **group** |  |  |  |  |  |
|  | Atezolizumab + EC | 100 (80.0) | 75 (78.1) | 25 (86.2) | 0.678 |
|  | Atezolizumab + EP | 11 (8.8) | 9 (9.3) | 2 (6.9) |  |
|  | Atezolizumab + EL | 6 (4.8) | 6 (6.3) | 0 (0.0) |  |
|  | Other | 8 (6.4) | 6 (6.3) | 2 (6.9) |  |
|  | Sum | 125 (100.0) | 96 (100.0) | 29 (100.0) |  |

**Abbreviations:** EP, Etoposide & Cisplatin; EC, Etoposide & Carboplatin; EL, Etoposide & Lobaplatin.

**Table S3.** Responses to the front-line chemotherapy or chemo-immunotherapy.

|  | **Total**  **(n=265)** | **The chemotherapy group (n=140)** | | |  | **The chemo-immunotherapy group (n=125)** | | |
| --- | --- | --- | --- | --- | --- | --- | --- | --- |
|  |  | **Central-type**  **(n=111)** | **Peripheral-type**  **(n=29)** | ***P* value** |  | **Central-type**  **(n=96)** | **Peripheral-type**  **(n=29)** | ***P* value** |
| **Response, No (%)** |  |  |  | 0.015 |  |  |  | 0.988 |
| Complete response | 10 (3.8) | 5 (4.5) | 2 (6.9) | 0.962 |  | 3 (3.1) | 0 (0.0) | 0.205 |
| Partial response | 175 (66.0) | 74 (66.7) | 11 (37.9) | 0.005 |  | 68 (70.8) | 22 (75.9) | 0.597 |
| Stable disease | 56 (21.1) | 22 (19.8) | 9 (31.0) | 0.195 |  | 19 (19.8) | 6 (20.7) | 0.916 |
| Progressive disease | 24 (9.1) | 10 (9.0) | 7 (24.2) | 0.057 |  | 6 (6.3) | 1 (3.4) | 0.909 |
|  |  |  |  |  |  |  |  |  |
| Objective response rate | 185 (69.8) | 79 (71.2) | 13 (44.8) | 0.008 |  | 71 (74.0) | 22 (75.9) | 0.837 |
| Disease control rate | 241 (90.9) | 101 (91.0) | 22 (75.9) | 0.026 |  | 90 (93.8) | 28 (96.6) | 0.909 |

**Table S4A.** Univariate and multivariate Cox regression analyses for PFS in the chemotherapy group.

| **Variable** | **Category** | **Univariate Analysis** | |  | **Multivariate Analysis** | |
| --- | --- | --- | --- | --- | --- | --- |
|  |  | **HR (95% CI)** | ***P* value** |  | **HR (95% CI)** | ***P* value** |
| **Age** | ≧65 y vs. <65 y | 0.78 (0.54-1.12) | 0.178 |  |  |  |
| **Gender** | Male vs. Female | 1.19 (0.68-2.08) | 0.553 |  |  |  |
| **ECOG PS** | 0~1 vs. 2 | 0.24 (0.06-1.00) | 0.050 |  | 0.40 (0.09-1.71) | 0.217 |
| **Smoking History** | Smokers vs. Non-smokers | 0.83 (0.55-1.25) | 0.373 |  |  |  |
| **Comorbidities** | Yes vs. No | 1.12 (0.77-1.62) | 0.563 |  |  |  |
| **Brain Metastasis** | Yes vs. No | 1.05 (0.71-1.55) | 0.804 |  |  |  |
| **Liver Metastasis** | Yes vs. No | 1.79 (1.10-2.92) | 0.019 |  | 1.91 (1.14-3.20) | 0.013 |
| **Bone Metastasis** | Yes vs. No | 1.66 (1.13-2.42) | 0.009 |  | 1.48 (1.00-2.18) | <0.050 |
| **Treatment Strategy** | EC vs. other | 0.63 (0.32-1.26) | 0.192 |  |  |  |
|  | EP vs. other | 0.74 (0.38-1.47) | 0.393 |  |  |  |
| **Tumor location** | Peripheral vs. Central | 2.23 (1.38-3.60) | 0.001 |  | 2.41 (1.46-3.99) | <0.001 |

**Abbreviations:** PFS, Progression-free survival; HR, Hazard ratio; CI, Confidence interval; ECOG PS, Eastern Corporation

Oncology Group performance status. EC, Etoposide & Carboplatin; EP, Etoposide & Cisplatin.

**Table S4B.** Univariate Cox regression analyses for OS in the chemotherapy group.

| **Variable** | **Category** | **HR (95% CI)** | ***P* value** |
| --- | --- | --- | --- |
| **Age** | ≧65 y vs. <65 y | 0.87 (0.57-1.33) | 0.507 |
| **Gender** | Male vs. Female | 1.67 (0.83-3.36) | 0.147 |
| **ECOG PS** | 0~1 vs. 2 | 0.49 (0.07-3.53) | 0.475 |
| **Smoking History** | Smokers vs. Non-smokers | 1.16 (0.70-1.91) | 0.559 |
| **Comorbidities** | Yes vs. No | 1.31 (0.85-2.01) | 0.227 |
| **Brain Metastasis** | Yes vs. No | 0.68 (0.43-1.10) | 0.117 |
| **Liver Metastasis** | Yes vs. No | 1.11 (0.57-2.17) | 0.763 |
| **Bone Metastasis** | Yes vs. No | 1.49 (0.96-2.32) | 0.073 |
| **Treatment Strategy** | EC vs. other | 0.82 (0.32-2.11) | 0.687 |
|  | EP vs. other | 0.70 (0.28-1.80) | 0.462 |
| **Tumor location** | Peripheral vs. Central | 1.34 (0.80-2.26) | 0.268 |

**Abbreviations:** OS, Overall survival; HR, Hazard ratio; CI, Confidence interval. EC,

Etoposide & Carboplatin; EP, Etoposide & Cisplatin.

**Table S4C.** Univariate Cox regression analyses for PFS in the chemo-immunotherapy group.

| **Variable** | **Category** | **HR (95% CI)** | ***P* value** |
| --- | --- | --- | --- |
| **Age** | ≧65 y vs. <65 y | 1.34 (0.85-2.10) | 0.208 |
| **Gender** | Male vs. Female | 0.72 (0.34-1.50) | 0.382 |
| **ECOG PS** | 0~1 vs. 2 | 0.79 (0.19-3.24) | 0.745 |
| **Smoking History** | Smokers vs. Non-smokers | 0.89 (0.57-1.39) | 0.596 |
| **Comorbidities** | Yes vs. No | 0.94 (0.58-1.54) | 0.809 |
| **Brain Metastasis** | Yes vs. No | 0.79 (0.40-1.53) | 0.478 |
| **Liver Metastasis** | Yes vs. No | 1.67 (0.99-2.79) | 0.053 |
| **Bone Metastasis** | Yes vs. No | 1.42 (0.84-2.38) | 0.192 |
| **Treatment Strategy** | Atezolizumab + EC vs. other | 1.29 (0.51-3.25) | 0.591 |
|  | Atezolizumab + EL vs. other | 2.14 (0.56-8.13) | 0.263 |
|  | Atezolizumab + EP vs. other | 0.77 (0.23-2.52) | 0.662 |
| **Tumor location** | Peripheral vs. Central | 0.71 (0.41-1.22) | 0.217 |

**Abbreviations:** PFS, Progression-free survival; HR, Hazard ratio; CI, Confidence interval; ECOG PS,

Eastern Corporation Oncology Group performance status. EC, Etoposide & Carboplatin; EL, Etoposide

& Lobaplatin; EP, Etoposide & Cisplatin.

**Table S4D.** Univariate Cox regression analyses for OS in the chemo-immunotherapy group.

| **Variable** | **Category** | **HR (95% CI)** | ***P* value** |
| --- | --- | --- | --- |
| **Age** | ≧65 y vs. <65 y | 1.44 (0.89-2.32) | 0.136 |
| **Gender** | Male vs. Female | 1.26 (0.51-3.13) | 0.620 |
| **ECOG PS** | 0~1 vs. 2 | 0.66 (0.16-2.73) | 0.566 |
| **Smoking History** | Smokers vs. Non-smokers | 1.05 (0.65-1.69) | 0.847 |
| **Comorbidities** | Yes vs. No | 1.04 (0.61-1.79) | 0.880 |
| **Brain Metastasis** | Yes vs. No | 0.84 (0.43-1.65) | 0.615 |
| **Liver Metastasis** | Yes vs. No | 1.83 (1.05-3.19) | 0.034 |
| **Bone Metastasis** | Yes vs. No | 1.23 (0.71-2.14) | 0.453 |
| **Treatment Strategy** | Atezolizumab + EC vs. other | 0.95 (0.58-1.55) | 0.835 |
|  | Atezolizumab + EL vs. other | 1.47 (0.49-4.38) | 0.489 |
|  | Atezolizumab + EP vs. other | 1.02 (0.52-1.99) | 0.951 |
| **Tumor location** | Peripheral vs. Central | 0.64 (0.35-1.18) | 0.156 |

**Abbreviations:** PFS, Progression-free survival; HR, Hazard ratio; CI, Confidence interval; ECOG PS,

Eastern Corporation Oncology Group performance status. EC, Etoposide & Carboplatin; EL, Etoposide

& Lobaplatin; EP, Etoposide & Cisplatin.

**Table S5.** Clinicopathological information of 9 patients included for scRNA-seq analyses.

| **ID** | **Gender** | **Age** | **Smoking history** | **Primary tumor location** |
| --- | --- | --- | --- | --- |
| P01 | Female | 51 | No | Peripheral |
| P02 | Male | 54 | Yes | Peripheral |
| P03 | Male | 65 | Yes | Central |
| P04 | Male | 70 | No | Peripheral |
| P05 | Male | 64 | Yes | Central |
| P06 | Male | 63 | Yes | Central |
| P07 | Male | 78 | Yes | Central |
| P08 | Male | 68 | Yes | Peripheral |
| P09 | Male | 65 | No | Central |
